# Structural and functional investigation of the DHH/DHHA1 family proteins in *Deinococcus radiodurans*

**DOI:** 10.1093/nar/gkae451

**Published:** 2024-05-28

**Authors:** Ying Wang, Wanshan Hao, Ziming Guo, Yiyang Sun, Yu Wu, Yukang Sun, Tianwen Gao, Yun Luo, Lizan Jin, Jieyu Yang, Kaiying Cheng

**Affiliations:** Zhejiang Key Laboratory of Medical Epigenetics, Department of Immunology and Pathogen Biology, School of Basic Medical Sciences, Affiliated Hospital of Hangzhou Normal University, Hangzhou Normal University, Hangzhou 311121, China; Zhejiang Key Laboratory of Medical Epigenetics, Department of Immunology and Pathogen Biology, School of Basic Medical Sciences, Affiliated Hospital of Hangzhou Normal University, Hangzhou Normal University, Hangzhou 311121, China; Zhejiang Key Laboratory of Medical Epigenetics, Department of Immunology and Pathogen Biology, School of Basic Medical Sciences, Affiliated Hospital of Hangzhou Normal University, Hangzhou Normal University, Hangzhou 311121, China; Zhejiang Key Laboratory of Medical Epigenetics, Department of Immunology and Pathogen Biology, School of Basic Medical Sciences, Affiliated Hospital of Hangzhou Normal University, Hangzhou Normal University, Hangzhou 311121, China; Zhejiang Key Laboratory of Medical Epigenetics, Department of Immunology and Pathogen Biology, School of Basic Medical Sciences, Affiliated Hospital of Hangzhou Normal University, Hangzhou Normal University, Hangzhou 311121, China; Zhejiang Key Laboratory of Medical Epigenetics, Department of Immunology and Pathogen Biology, School of Basic Medical Sciences, Affiliated Hospital of Hangzhou Normal University, Hangzhou Normal University, Hangzhou 311121, China; Zhejiang Key Laboratory of Medical Epigenetics, Department of Immunology and Pathogen Biology, School of Basic Medical Sciences, Affiliated Hospital of Hangzhou Normal University, Hangzhou Normal University, Hangzhou 311121, China; Zhejiang Key Laboratory of Medical Epigenetics, Department of Immunology and Pathogen Biology, School of Basic Medical Sciences, Affiliated Hospital of Hangzhou Normal University, Hangzhou Normal University, Hangzhou 311121, China; Zhejiang Key Laboratory of Medical Epigenetics, Department of Immunology and Pathogen Biology, School of Basic Medical Sciences, Affiliated Hospital of Hangzhou Normal University, Hangzhou Normal University, Hangzhou 311121, China; Zhejiang Key Laboratory of Medical Epigenetics, Department of Immunology and Pathogen Biology, School of Basic Medical Sciences, Affiliated Hospital of Hangzhou Normal University, Hangzhou Normal University, Hangzhou 311121, China; Zhejiang Key Laboratory of Medical Epigenetics, Department of Immunology and Pathogen Biology, School of Basic Medical Sciences, Affiliated Hospital of Hangzhou Normal University, Hangzhou Normal University, Hangzhou 311121, China; State Key Laboratory for Diagnosis and Treatment of Infectious Diseases, The First Affiliated Hospital, College of Medicine, Zhejiang University, Hangzhou 310003, China

## Abstract

DHH/DHHA1 family proteins have been proposed to play critical roles in bacterial resistance to environmental stresses. Members of the most radioresistant bacteria genus, *Deinococcus*, possess two DHH/DHHA1 family proteins, RecJ and RecJ-like. While the functions of *Deinococcus radiodurans* RecJ (DrRecJ) in DNA damage resistance have been well characterized, the role and biochemical activities of *D. radiodurans* RecJ-like (DrRecJ-like) remain unclear. Phenotypic and transcriptomic analyses suggest that, beyond DNA repair, DrRecJ is implicated in cell growth and division. Additionally, DrRecJ-like not only affects stress response, cell growth, and division but also correlates with the folding/stability of intracellular proteins, as well as the formation and stability of cell membranes/walls. DrRecJ-like exhibits a preferred catalytic activity towards short single-stranded RNA/DNA oligos and c-di-AMP. In contrast, DrRecJ shows no activity against RNA and c-di-AMP. Moreover, a crystal structure of DrRecJ-like, with Mg^2+^ bound in an open conformation at a resolution of 1.97 Å, has been resolved. Subsequent mutational analysis was conducted to pinpoint the crucial residues essential for metal cation and substrate binding, along with the dimerization state, necessary for DrRecJ-like's function. This finding could potentially extend to all NrnA-like proteins, considering their conserved amino acid sequence and comparable dimerization forms.

## Graphical Abstract

**Graphical Abstract:**
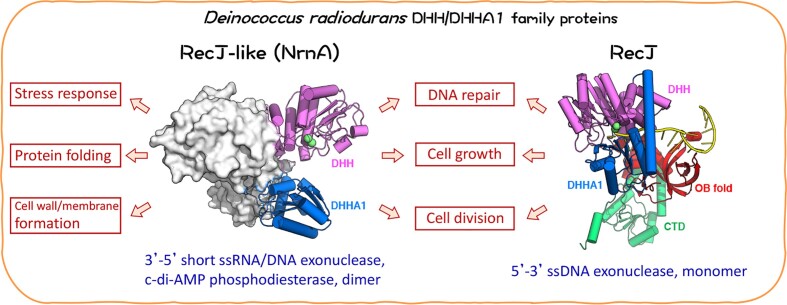

## Introduction

Bacteria encode multiple proteins to overcome survival stress arising from nutritional deficiency, DNA damage, or the accumulation of harmful metabolites. RNA degradation plays a crucial role in overall RNA metabolism and significantly impacts intracellular RNA levels (1). However, the degradation of RNA by degradosomes typically yields nanoRNAs, short RNA fragments ranging from 2 to 5 nucleotides in length, rather than complete degradation to single nucleotides (1,2). These nanoRNAs can act as primers for transcription initiation and potentially disrupt global gene expression, inducing metabolic stress and ultimately leading to cell death (3). Therefore, the complete degradation of nanoRNAs is essential for maintaining genomic integrity and cell survival. These nanoRNAs can be digested by oligoribonuclease (Orn), whose function and structure have been extensively studied in *Escherichia coli, Pseudomonas aeruginosa, Vibrio cholerae, Coxiella burnetii, Colwellia psychrerythraea 34H*, etc. (4–8). NanoRNase (Nrn) serves as a functional homolog of Orn and is widely distributed in bacteria and archaea where Orn is absent (9). There are three types of NanoRNases (Nrn A, B and C), with little sequence homology identified among them. NrnC, evolved from RNase D, exhibits exquisite substrate preference for diribonucleotides (6,10). NrnA and NrnB belong to the DHH/DHHA1 phosphoesterase superfamily, featuring a conserved DHH motif (located in the DHH domain) and a conserved GGGH motif (located in the DHHA1 domain) in their active site (11–13).

NrnA was initially identified in *Bacillus* species and has been demonstrated to possess exonuclease activity on nanoR(D)NAs, hydrolytic capability on adenosine 3′, 5′-bisphosphate (pAp) similar to CysQ, and cyclic nucleotide phosphodiesterase activity (11,14,15). It has been observed that NrnA exhibits a preference for small nucleotides but can also act on longer oligonucleotides (16,17). NrnA can directly hydrolyze c-di-A(G)MP to 5′-pA(G) via the intermediate 5′-pA(G)pA(G) (11,14,15). However, other cyclic nucleotide phosphodiesterases, such as GdpP, PgpH and CdnP, specifically catalyze the breakdown of c-di-A(G)MP to 5′-pA(G)pA(G) (18–20). GdpP belongs to the DHH/DHHA1 phosphoesterase superfamily but also contains transmembrane helices, a PAS domain, and a degenerate GGDEF domain, in addition to the DHH/DHHA1 domains. PgpH and CdnP belong to another cyclic nucleotide phosphodiesterase family that lacks DHH-DHHA1 domains (21). Moreover, AtaC is a recently identified noncanonical cyclic nucleotide phosphodiesterase that can hydrolyze c-di-AMP first to pApA and subsequently to AMP, similar to NrnA, but with a different domain arrangement (22). Deletion of the *nrnA* gene led to cell growth and stress tolerance defects in *Streptococcus pneumoniae* and *Streptococcus aureus* (14–16,23). Whether NrnA plays a (partial) backup role, akin to the DNA repair protein RecJ, another DHH/DHHA1 exonuclease, remains unknown. On the other hand, NrnB has been identified as a second nanoRNase of *B. subtilis*, with its substrate strictly restricted to nanoRNA (12).

*Deinococcus radiodurans*, renowned for its remarkable resistance to DNA damage, possesses a highly efficient DNA damage response and repair system (24,25). *D. radiodurans* harbors a homolog of CysQ (DR_1703) but lacks homologs of Orn, GdpP, PgpH, CdnP and AtaC (26). Additionally, *D. radiodurans* possesses two DHH/DHHA1 family homologs, namely DR_1126 and DR_0826, annotated as RecJ and RecJ-like, respectively. While the structure, function, and molecular mechanisms of DrRecJ have been extensively studied (27,28), information regarding the structural and functional characteristics of DrRecJ-like remains elusive.

In this study, we constructed *drrecJ-like* single and *drrecJ*/*drrecJ-like* double knockout strains, examining their phenotypes under various stress conditions and transcriptomic differences during normal growth at 30°C. The biochemical properties and crystal structure of DrRecJ-like were characterized, and key residues for metal cation and substrate binding were predicted and subsequently confirmed through point mutations. Furthermore, the functional significance of DrRecJ-like dimerization was investigated.

## Materials and methods

### Cloning and site-directed mutagenesis

All primers and oligonucleotides utilized in this study were procured from Generay Biotech (Shanghai, China) and are listed in Supplementary Table S1. *Escherichia coli* and *D. radiodurans* strains were cultured and transformed following previously described protocols (28).

For constructing the DrRecJ-like expression vector, the full-length gene (DR_0826) encoding DrRecJ-like (residues 1–338 aa) was amplified from *D. radiodurans* genomic DNA and inserted into a modified pET28a expression vector, pET28-HMT. This vector includes a fused N-terminal 6 × His tag, an MBP-tag, and a TEV protease recognition site (His-MBP-TEV). The expression vector for DrCysQ was generated by ligating the *drcysQ* gene (DR_1703) into the pET28a expression vector. These vectors were transformed into the *E. coli* DH5α strain (TransGen Biotech, Beijing), selected from kanamycin-containing plates (50 μg/ml), and further validated through sequencing.

To construct the complementation plasmid, the *drrecJ-like* gene or its derivatives were cloned into the NdeI and BamHI sites of the shuttle vector pRADK. The resulting vectors were then transformed into the *E. coli* DH5α strain, selected from ampicillin-containing plates (100 μg/ml), and subsequently confirmed by sequencing.

Site-directed mutagenesis was performed using the QuikChangeTM Site-Directed Mutagenesis Kit from Stratagene (La Jolla, CA), as previously described (28). The fidelity of the constructed vectors was confirmed through sequencing. All successfully constructed expression vectors were transformed into the *E. coli* Rossetta (DE3) strain (TransGen Biotech, Beijing).

### Mutant strain construction and complementation

The *D. radiodurans* wild-type (WT) strain R1 and its derivatives were cultivated at 30°C either in TGY broth (0.5% tryptone, 0.1% glucose, and 0.3% yeast extract) or on TGY agar plates (TGY broth with 1.25% agar). For the transformation of *D. radiodurans* cells, a modified CaCl_2_ technique described previously was utilized (29). *D. radiodurans* cells were cultured to the early stationary phase (OD_600_= 0.6–0.8). The cells were harvested, washed twice with 2 × TGY medium (with 30 mM CaCl_2_), and resuspended in 500 μl TGY medium (with 30 mM CaCl_2_) and incubated at 30°C for 1 h. Aliquots containing plasmid or DNA fragments (200 ng) were added and incubated on ice for an additional 1 h. Five milliliters of fresh TGY medium was added to the aliquot, and the mixture was continuously incubated at 30°C for 24 h, followed by plating on TGY plates.

The knockout of *drrecJ-like* or *drrecJ-like/drrecJ* was performed using a deletion replacement method as previously described (29). Upstream and downstream sequences of the *drrecJ-like* gene with BamHI and HindIII digestion sites, respectively, were amplified. After BamHI and HindIII enzyme digestion, these segments were ligated to a kanamycin resistance cassette (*Kan^r^*) (with BamHI and HindIII enzyme digestion as well) and transformed into the wild-type *D. radiodurans* strain R1 or *drrecJ* knockout strain △*J*. The deletion mutant strains (named △*J-like* and △*J/*△*J-like*) were screened on kanamycin-containing (10 μg/ml) TGY plates and confirmed by sequencing. To construct the complementary strains, shuttle vectors pRADK, harboring the genes *drrecJ* or *drrecJ-like*, were transformed into the mutant strains following previously established procedures (30). The complementary strains (named △*J-like + J-like* or △*J + J*) were selected on chloromycetin-containing (4 μg/ml) TGY plates.

### Growth curve assays

Growth curve and temperature-sensitive assays were conducted using previously described methods (30). Briefly, when the cell density of the culture (OD_600_) reached 1.0, 1-ml aliquots were resuspended in 100 ml of new fresh TGY medium and incubated at either 30°C or 37°C. The cell growth rate was monitored by measuring the OD_600_ at various incubation times (every hour, until 60 h). Three replicates were performed for each strain. Growth curves were generated using GraphPad Prism 9.0.

### DNA-damaging agent and detergent survival rate assays

Survival rate assays were conducted following previously established protocols (27,29,30). Cells were cultivated in TGY medium supplemented with the appropriate antibiotics to reach the early exponential phase (OD_600_= 1.0). For UV treatment, cells were diluted to suitable concentrations and spotted on TGY plates. After complete absorption, the plates were exposed to UV at dose of 400 J/m^2^. For hydrogen peroxide (H_2_O_2_) treatment, after a series of dilutions with 1 × autoclaved phosphate-buffered saline (PBS) buffer, cultures were treated with 50 mM H_2_O_2_ for 30 min. Before being spotted onto TGY plates, residual H_2_O_2_ was diluted with excess catalase. Cells without H_2_O_2_ treatment served as controls. For mitomycin C (MMC) treatment, cells were incubated with 40 μg/ml of MMC at 30°C for 20 min, washed with 1 × PBS buffer, serially diluted 10-fold at each step, and spotted onto TGY agar plates. For detergent treatment, cells were incubated with 0.1% tween 20 at 30°C for 30 min, washed with 1 × PBS buffer, serially diluted 10-fold at each step, and spotted onto TGY agar plates. Plates were cultured for 2−3 days at 30°C or 37°C.

### Extraction of total RNA from *D. radiodurans*

Wild-type and mutant cells were cultured until reaching an OD_600_ of 0.8. They were then centrifuged and resuspended in 1 × PBS buffer at pH 7.0. Total RNA was extracted from two independent biological replicates for each group using the TRIzol method following the manufacturer's protocol (Ambion, Foster City, CA, USA). RNA quality, including degradation and contamination, was assessed by electrophoresis on 1% agarose gels. Additionally, RNA integrity was evaluated using the RNA Nano 6000 Assay Kit on the Bioanalyzer 2100 system (Agilent Technologies, CA, USA).

### Transcriptome library preparation, clustering and sequencing

Transcriptome library preparation, clustering and sequencing were performed by Novogene Corporation (Beijing, China).

Two micrograms of RNA per sample was used as the input material for library preparation, and then, a Ribo-Zero™ Magnetic Kit (Epibio, MRZB12424) was used to remove rRNA. Subsequently, the obtained mRNA was randomly fragmented using divalent cations in the Fragmentation Buffer. Using the fragmented mRNA as a template and random oligonucleotides as primers, the first strand of cDNA was synthesized in the presence of MMuLV reverse transcriptase (NEB, USA). Then, the RNA strand was degraded by RNase H (NEB, USA), and the second strand of cDNA was synthesized using dUTP instead of dTTP as the raw material in the presence of DNA polymerase I. The purified double-stranded cDNA underwent end repair, adenylation, and adapter ligation. USER enzyme (NEB, USA) was then added to degrade the second cDNA strand containing uracil (U). The cDNA fragments of approximately 370–420 bp were size-selected using AMPure XP beads, followed by PCR amplification. The PCR products were purified again using AMPure XP beads to obtain the final library.

Following library construction, initial quantification was conducted using the Qubit 2.0 Fluorometer. The library was then diluted to a concentration of 1.5 ng/μl. Subsequently, the insert size of the library was assessed using the Agilent 2100 bioanalyzer. Once the insert size met expectations, qRT-PCR was utilized for accurate quantification of the library's effective concentration, ensuring an effective concentration above 2nM to maintain library quality.

Upon passing quality control, different libraries were pooled based on their effective concentrations and the desired amount of data for sequencing on the Illumina platform, generating 150 bp paired-end reads. The sequencing process operates on the principle of Sequencing by Synthesis, where four fluorescently labeled dNTPs are incorporated into the flow cell during synthesis and sequencing. DNA polymerase, along with adapter primers, amplifies the DNA. As each complementary strand extends in every sequencing cluster, the addition of a fluorescently labeled dNTP releases a corresponding fluorescence signal. The sequencer captures these signals, and with the assistance of computer software, converts them into sequencing peaks to obtain the sequence information of the target fragment.

### Transcriptomic analysis by RNA-seq

After filtering the raw data, conducting sequencing error rate checks, and examining GC content distribution, clean reads were obtained for subsequent analysis. The filtered sequencing reads were subjected to genome mapping analysis using the Bowtie2 software (Langmead, B. 2012). Based on the positional information of gene alignments on the reference genome, the number of reads covering each gene (including newly predicted genes) within the start and end range is tallied. Reads with alignment quality scores below 10, unpaired alignments, and reads aligning to multiple regions of the genome are subsequently filtered out.

After quantifying gene expression, to identify genes with significantly different expression levels across different conditions, statistical analysis on the expression data was performed. Differential expression analysis was performed using the software DESeq2 (https://www.bioconductor.org/packages/2.13/bioc/html/DESeq.html), which provides statistical routines for determining differential expression in digital gene expression data using a negative binomial distribution model. Genes with |log_2_(FoldChange)| > 0 and *P*_adj_ < 0.05 were considered significantly differentially expressed. Gene KEGG pathway analyses were used to identify the enriched molecular functions and associated biological pathways of DEGs. The enrichment analyses were carried out through software clusterProfiler (31). Only terms with a *P*_adj_ < 0.05 were considered to be significant and be analyzed.

### Protein expression and purification

The protein expression and purification of tag-free DrRecJ-like and mutants were conducted according to the reference (28), with some modifications. In brief, transformed *E. coli* Rossetta (DE3) clones were cultured at 37°C to an optical density of OD_600_ (0.6–0.8) in LB medium supplemented with 50 μg/ml kanamycin. Protein expression was induced at 18°C for 18 h by adding isopropyl-b-d-thiogalactopyranoside (IPTG) to a final concentration of 0.4 mM. After harvesting, cells were re-suspended in lysis buffer (20 mM Tris (pH 7.5), 100 mM NaCl, 5% (w/v) glycerol, 1 mM Tris(2-carboxyethyl) phosphine (TCEP), and 1 mM Ethylene Diamine Tetra acetic Acid (EDTA)), lysed by sonication, and centrifuged at 18 000 g for 30 min at 4°C. The supernatant was purified using a cOmplete His-Tag Purification Column (Roche, Switzerland), which was equilibrated with buffer A (20 mM Tris (pH 7.5), 100 mM NaCl, 5% (w/v) glycerol, 0.5 mM TCEP, and 1 mM EDTA), washed with 1 mM imidazole and finally eluted with 300 mM imidazole. The eluted fractions were subsequently loaded onto a HiTrap Q column (GE Healthcare) pre-equilibrated with buffer B (20 mM Tris (pH 7.5), 100 mM NaCl, 1 mM EDTA, 5% (w/v) glycerol and 1 mM TCEP). Fractions containing tagged DrRecJ-like protein were eluted using a linear gradient of 100–600 mM NaCl. After TEV-tag-removal using TEV protease, the protein was reloaded onto the cOmplete His-Tag Purification Column to remove the uncleaved protein and TEV protease. The flow-through fractions were collected and concentrated. Proteins were finally purified by Superdex 75 10/300 GL column (GE Healthcare) using buffer B. Each fraction was analyzed by 12% sodium dodecyl sulfate polyacrylamide gel electrophoresis (SDS-PAGE). Fractions containing the purified proteins were pooled, concentrated, flash-frozen in liquid nitrogen, and stored at − 80°C.

The protein expression and purification of tag-free DrRecJ followed the protocol outlined in reference (28).

The protein expression of DrCysQ was performed in a manner similar to that of DrRecJ-like. Cells were harvested and lysed. The supernatant was purified using a cOmplete His-Tag Purification Column (Roche, Switzerland), which was equilibrated with buffer A, washed with 1 mM imidazole and finally eluted with 300 mM imidazole. The eluted fractions were subsequently loaded onto a HiTrap Q column (GE Healthcare) pre-equilibrated with buffer B. The flow-through fractions were collected and concentrated. Proteins were finally purified by Superdex 75 10/300 GL column (GE Healthcare) using buffer B.

### Analytical gel filtration analysis

The dimerization of DrRecJ-like was investigated using analytical gel filtration, following previously established protocols with slight modifications (32). In brief, 300 μl of gel filtration buffer (20 mM Tris (pH 8.0), 100 mM NaCl and 1 mM TCEP) containing DrRecJ-like (1 mM) was centrifuged at 18000 g for 5 min to remove any precipitated protein. Subsequently, the supernatant was loaded onto a Superdex 75 10/300 GL column (GE Healthcare). The sizes of calibration proteins, indicated by their elution positions, were annotated on the *x*-axis using the method outlined in the gel filtration calibration kit HMW (GE Healthcare).

### Crystallization and structure determination

Crystallization trials were performed using the sitting drop vapor diffusion method at 289 K. In brief, the freshly purified protein was concentrated to ∼30 mg/ml and centrifuged to remove insoluble fractions before crystallization. After a series of screening tests and optimizations, the best crystals of DrRecJ-like alone were obtained under the condition of 1.4 M MgSO_4_, 0.08 M MES (pH 6.5) and 15% (v/v) glycerol. Cryocooling was achieved by stepwise soaking of the crystals in the reservoir solution containing 10, 20 and 30% (w/v) glycerol for 1 min, followed by flash freezing in liquid nitrogen. X-ray diffraction data were collected on beamline BL02U1 at Shanghai Synchrotron Radiation Facility (Shanghai, China) and integrated and scaled by Aquarium pipeline (33,34). The apo structure of DrRecJ-like was determined by the molecular replacement method using the *Thermotoga maritima* PDE apo structure (PDB code: 5O1U) as the search model. Structures were refined using PHENIX (35), and interspersed with manual model building using COOT (36). All residues were in the most favorable and allowed regions of the Ramachandran plot. The structural figures were created using PyMOL. The statistics for the data collection and refinement are listed in Table 2.

### Nuclease activity assays

The nuclease activity assays were conducted following established protocols (27). All oligonucleotides were obtained from Generay (Shanghai, China) and labeled with 6-carboxfluorescein (6-FAM) at the 3′ or 5′ end. The oligonucleotide sequences are provided in Table S1. For a typical nuclease assay, 100 nM (or 200 nM in some assays) of ssDNA or ssRNA was incubated with varying concentrations (6.25–500 nM) of freshly prepared wild-type or mutated DrRecJ-like proteins in a 10 μl reaction volume containing 50 mM Tris (pH 7.5), 100 mM NaCl, 0.1 mg/mL BSA, 1 mM DTT, 5% (v/v) glycerol, and 5 mM MnCl_2_ at 37°C for 30 min. The reactions were stopped with 2 × stop buffer (10 mM EDTA, 98% formamide) and incubated at 100°C for 10 min, followed by rapid cooling on ice. Reaction products were resolved on 15% polyacrylamide gels containing 7 M urea. The gels were imaged in fluorescence mode (FAM) using ChemiScope6100 (Clinx Science Instruments, Shanghai), and bands were analyzed with Image J Software (National Institutes of Health, USA). To assess metal preference, 100 nM 20 nt 5′FAM labeled ssRNA was incubated with 500 nM DrRecJ-like, in the presence of 10 mM of EDTA, MnCl_2_, CaCl_2_, CoCl_2_, MgCl_2_, NiCl_2_ or ZnCl_2_. For the divalent metal ion concentrations test, 100 nM 20 nt 5′FAM labeled ssRNA was incubated with 100 nM DrRecJ-like (when MnCl_2_ or CoCl_2_ was used), or 200 nM DrRecJ-like (when NiCl_2_ was used), or 500 nM DrRecJ-like (when MgCl_2_ was used). Different concentrations (0, 0.25, 0.5, 1.0, 2.0, 4.0 and 8.0 mM) of divalent metal cations was introduced. For the determination of optimum substrate lengths and sequence, 0.8 mM substrates (pApA, pApApA, pApApApA, pApApApApA, 20 nt poly A, pUpUpUpUpU, pCpCpCpCpC and pGpGpGpGpG) were incubated with 0.5 μM wild-type DrRecJ-like in 5 μl reaction volumes containing 50 mM Tris (pH 8.0), 100 mM NaCl, 1 mM DTT, and 1 mM MnCl_2_ at 37°C for various durations (0, 5, 10, 15, 20, 30 min). The resulting products were subsequently separated using thin-layer chromatography (TLC) as outlined below. The digestion fractions were calculated by Image J from three replicates, and displayed as line chart or columns using GraphPad Prism 9.

### The TLC analysis for phosphodiesterase activity

For a standard assay, 0.8 mM c-di-AMP was incubated with 2.0 μM wild-type DrRecJ-like in a 5 μl reaction volume containing 50 mM Tris (pH 8.0), 100 mM NaCl, 1 mM DTT, and 1 mM MnCl_2_ at 37°C for various durations (0, 5, 10, 15, 20, 30 min). The reactions were quenched with 2 × stop buffer. Enzymatic digestion products were resolved by TLC on silica gel plates (Macklin, China) in solvent A (saturated ammonium sulphate/3M sodium acetate/iso-propanol; 80:6:2) as previously described (37). The reaction products and nucleotide standards were visualized under UV light (365 nm).

### Inorganic phosphate detection assay

The digestion efficiency of pAp, pApA and ApAp was assessed by spectrophotometric detection of inorganic phosphate (Pi) using the phosphomolybdate blue reagent. The inorganic phosphate assay kit was obtained from Sangon (Shanghai, China). Reaction mixtures (5 μl) containing 200 μM DrRecJ or DrRecJ-like, or 2 μM DrCysQ, 200 μM pAp and 1 mM MnCl_2_, were incubated in the reaction buffer (50 mM Tris, pH 8.0; 100 mM NaCl; and 1 mM DTT) at 37°C for 20 min. Subsequently, 95 μl of diluted phosphomolybdate blue reagent was added, and Pi release was monitored at 660 nm using a microplate reader (ALLSHNEG, Hangzhou). The blank control was established in the absence of pAp and proteins, and this result served as the background measure, normalized to zero. The negative-control experiment was conducted in the presence of 200 μM pAp without proteins, or with the DrRecJ-like nuclease-dead mutant, DrRecJ-likeD95A. Proteins alone were confirmed not to influence the OD values. Standard Pi reagent (200 μM) was included as a positive control. The OD_660nm_ values of three replicates were presented as columns using GraphPad Prism 9.

### The *in vivo* oligonucleotide content detection assay

The *in vivo* accumulation of oligonucleotides was detected using an oligonucleotide ELISA assay kit (KESHUN BIO, China). Wild-type or mutant cells (250 ml) were cultured until reaching an OD_600_ of 1.0. The cells were then centrifuged, washed with pre-chilled 1 × PBS buffer four to five times, and resuspended in 2 ml of pre-chilled buffer T (containing 0.3 mg/ml lysozyme, 20 mM EDTA, 0.5 mM TCEP, 0.1 U/μl RNAse inhibitor, and 1 × PBS buffer). Subsequently, the cells were sonicated and centrifuged at 18000 g for 10 min. The supernatant of each sample was collected, and samples were normalized to the same concentration using buffer T based on their total protein concentrations. Next, an oligonucleotide ELISA assay kit (KESHUN BIO, China) was used. Ten microliters of each sample were mixed with 40 ul of diluent and 50 ul of HRP-conjugate reagent in the well of the ELISA plate. After aspirating each well and washing with 400 μl of Wash Solution four times, the remaining Wash Solution was completely removed. Then, 50 μl of chromogen solution A and B were added to each well, gently mixed, and incubated at 37°C for 15 min. The OD_450_ value was recorded using a microplate reader (ALLSHNEG, Hangzhou) within 15 min. A series of standard samples were used to generate the standard curve, and the concentrations of oligonucleotides for each strain were calculated according to the standard curve. Three replicates were conducted.

## Results

### Deletion of *drrecJ-like* results in growth defect and reduced stress resistance

**Figure 1.**
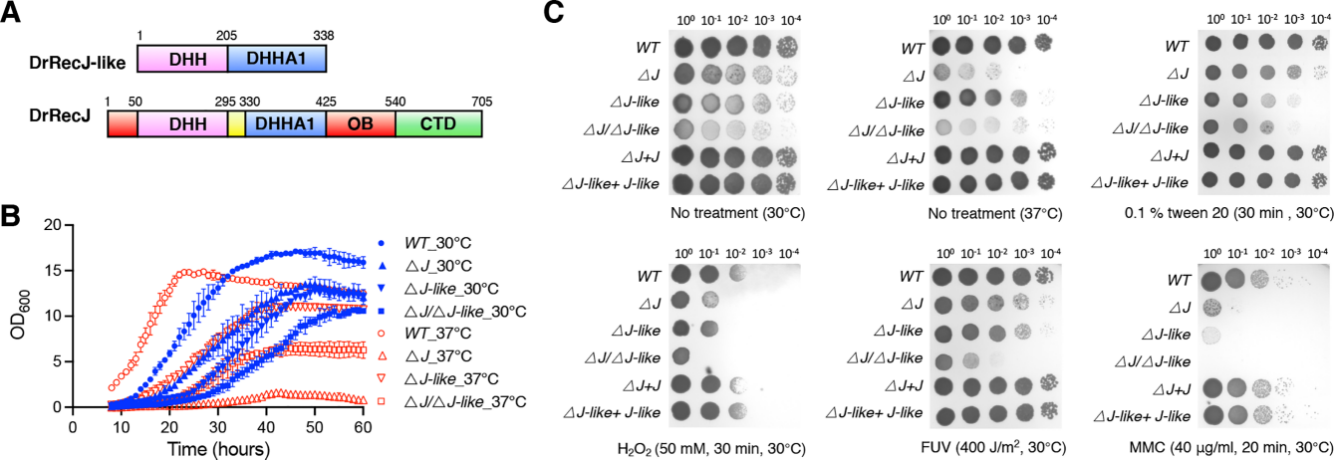
Domain arrangement and phenotypes of deletion mutant and complementation strains. (**A**) Schematic representation of the domain arrangements of DrRecJ-like and DrRecJ. (**B**) Growth curves of the wild-type strain R1 (WT), *drrecJ* single deletion mutant (*△J*), *drrecJ-like* single deletion mutant (*△J-like*), and the *drrecJ-like/recJ* double deletion mutant (*△J/△J-like*) at 30 and 37 degrees. (**C**) The WT, *△J*, *△J-like*, *△J/△J-like* and related complementation strains (*△J + J* and *△J-like + J-like*) were treated with 0.1% tween 20 (for 30 min), 50 mM H_2_O_2_ (for 30 min), FUV (for 400 J/m^2^), or 40 μg/ml MMC (for 30 min). Cells were diluted and spotted on plates. Plates were cultured for 2−3 days at 30°C or 37°C.

Through sequence homology searches, we identified two DHH/DHHA1 family proteins in *D. radiodurans*, RecJ protein (DR_1126) and RecJ-like protein (DR_0826). DrRecJ harbors a DHH domain, a DHHA1 domain, an OB fold domain and a C-terminal domain (Figure 1A). As previously reported, all domains of DrRecJ collectively endow it as a 5′-3′ exonuclease, with a high degree of processivity (27,28). In contrast, DrRecJ-like only harbors a DHH and a DHHA1 domain and its biochemical features and biological functions have not been studied (Figure 1A).

To investigate the biological function of DrRecJ-like, *drrecJ-like* gene was knocked out by deletion replacement. A *drrecJ-like/recJ* double deletion mutant (*△J/△J-like*) was generated by deleting the *drrecJ-like* gene based on the *drrecJ* deletion mutant (*△J*). All mutants were verified by PCR and sequencing (Supplementary Table S1 and Supplementary Figure S1). The complementary strains (named *△J-like + J-like* or *△J + J*) were generated by transforming the shuttle vectors ligated with respective genes.

To monitor the cell growth, the OD_600_ value for each strain was measured hourly for 60 h. Compared with the wild-type strain R1 (WT), *drrecJ-like* single deletion mutant (*△J-like*), *△J* and *△J/△J-like* exhibited an obvious growth defect at 30°C (Figure 1B). Interestingly, deletion of DrRecJ-like could partially repress the growth defect of *△J* mutant under 37°C. To investigate the function of the DrRecJ-like protein in the stress response process, the survival rates of *D. radiodurans* cells after treatment with detergent or various DNA-damaging agents were analyzed. Compared with the wild-type strain, both *△J-like* and *△J/△J-like* exhibited approximately a 10-fold reduction in resistance to 0.1% tween 20, while *△J* has no effect. Similar to *△J*, *△J-like* was also sensitive to H_2_O_2_ (50 mM) and mitomycin-C (MMC, 40 μg/ml) treatments (Figure 1C), whereas the *△J/△J-like* double mutant showed additive effects. Neither *ΔJ* nor *ΔJ-like* single mutants were sensitive to FUV treatment (400 J/m^2^), but *ΔJ/ΔJ-like* exhibited approximately a 100-fold reduction in resistance. Complementation of related genes could restore the defects.

These phenotypes indicate that DrRecJ-like plays important roles in stress response processes, such as DNA damage stress and cell membrane stress. However, RecJ and RecJ-like proteins might play similar roles in some pathways but have a mutually inhibitory effect on certain pathways.

### Transcriptomic analysis of *drrecJ-like* and *drrecJ* mutant strains

To elucidate the intricate phenotypic outcomes observed earlier, we performed a transcriptomic analysis of WT, *△J*, *△J-like* and *△J/△J-like* strains under normal growth condition at 30°C, without any treatment. Bacterial cultures were harvested during the early-logarithmic phase (OD_600_= 0.8), and total RNA was extracted for subsequent RNA sequencing. Three paired-end libraries were generated, providing an average of 2.3 G clean bases data per sample, which corresponded to 96.40–98.95% of the mapped genome of *D. radiodurans* (Supplementary Table S2).

**Figure 2.**
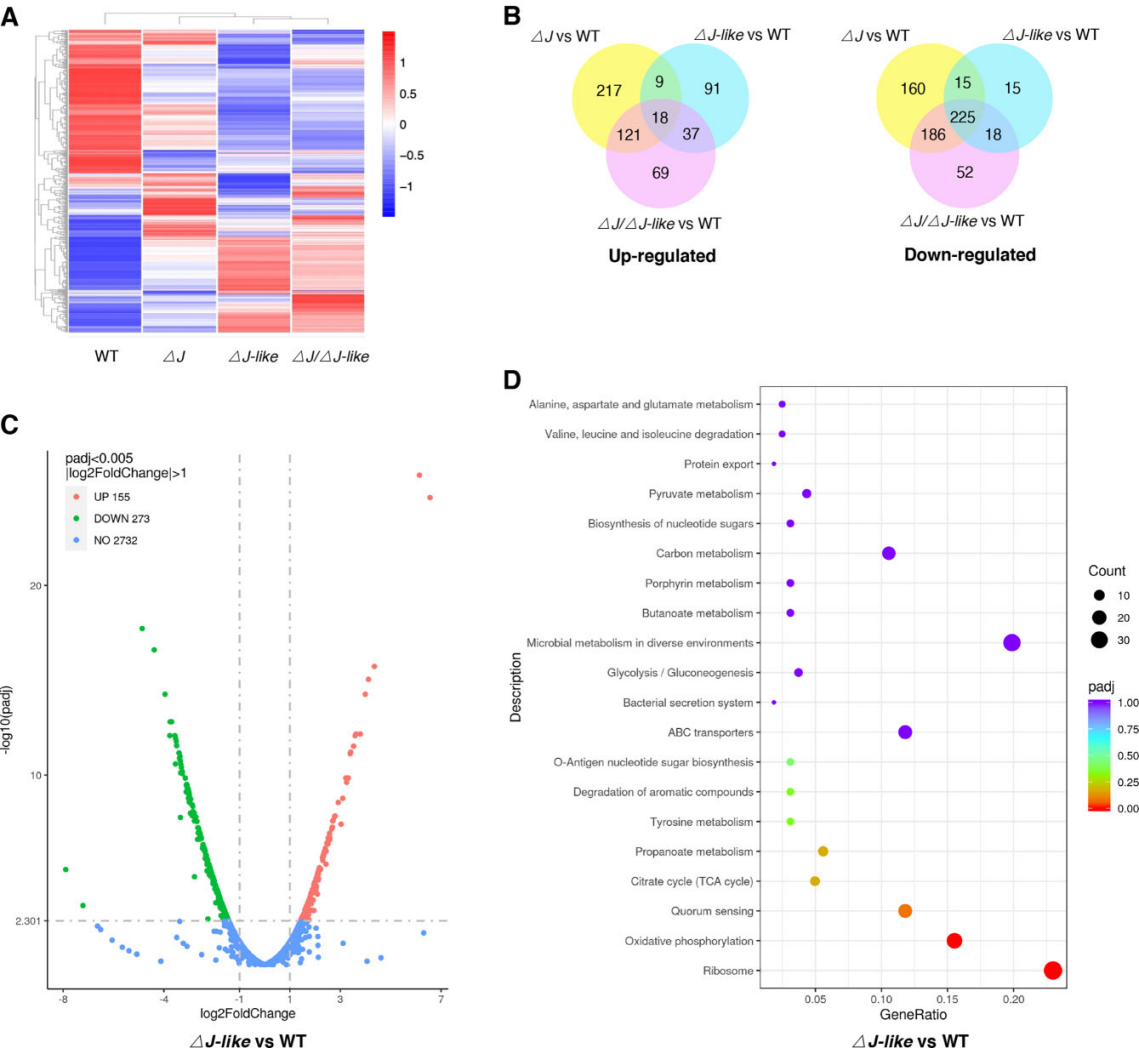
Transcriptome analysis by RNA-seq. (**A**) Clustered heatmap showing differential gene expressions (DGEs) in WT, *△J*, *△J-like*, and *△J/△J-like* mutant strains. (**B**) Venn diagram analysis of up-regulated (left) and down-regulated (right) DEGs in *△J* versus WT strain (yellow), △J-like versus WT strain (cyan), and △J /△J-like mutants versus WT strain (pink). (**C**) Volcano plot analysis showing DEGs profile between △J-like and WT strain. The gray dashed line indicates |log_2_ fold change| > 1 and adjusted *P*-value < 0.005. (**D**) The top20 KEGG enrichment pathways for DEGs in pairwise comparison of △J-like versus WT. The rich factor refers to the ratio of the number of DEGs in the pathway and the number of all annotated genes in the pathway.

Hierarchical clustering heatmap, Venn diagram, Volcano plot analysis, and top 20 KEGG enrichment pathways of the whole-genome transcriptomes for both the wild-type strain and the three knockout strains revealed significant profiles of differentially expressed genes (DEGs) (Figure 2; Supplementary Figure S2).

The Venn diagram illustrates that, among all the upregulated genes, only 18 genes are commonly shared among the three mutant strains, including DNA damage response protein DdrA (DR_0423) and DdrC (DR_0003) (Figure 2B; Supplementary Table S3). Conversely, among all the downregulated genes, 225 genes are commonly shared among the three mutant strains, including 33 ribosomal proteins, some transcription/translation/cell division-related proteins, and numerous quorum sensing and oxidative phosphorylation-associated proteins. This explains why these mutant strains all exhibit slowed growth and are sensitive to some DNA damage reagents such as H_2_O_2_ and MMC.

Among the upregulated and downregulated genes, 121 and 186 genes, respectively, are shared between both the *ΔJ* and *ΔJ/ΔJ-like* strains, but absent in the *ΔJ-like* strain (Figure 2B). The expression of some DNA damage response and repair-related genes was only significantly influenced by DrRecJ in this study, such as uracil-DNA glycosylase (DR_1751), DNA recombination-mediator protein A (DprA, DR_0120), and DNA damage response modulator PprM (DR_0907). Interestingly, despite RecJ being established as a DNA repair-related protein, its absence resulted in the upregulation/downregulation of genes which are mostly associated with DNA replication, transcription, translation, cell division, and cell metabolism (Table 1; Supplementary Table S3), aligning with the phenotype of slowed growth (or halted growth at 37°C) observed in the strains absence of RecJ.

**Table 1.** Selected genes of interest from transcript level analysis of RNA-seq

gene_id^a^	gene_id^b^	gene_name/description	△J-like*	△J*	△J/△J-like*
**Genes that are up/down-regulated simultaneously in both *△J* and *△J-like*, but *△J/△J-like* strain**					
A2G07_RS06990	DR_1306	hypothetical protein	2.53	1.72	NA
A2G07_RS06355	DR_1439	heme-binding domain-containing protein	4.12	1.56	NA
A2G07_RS06260	DR_1459	S8 family serine peptidase	2.13	1.60	NA
A2G07_RS10455	DR_0604	Acetyltransferase (GNAT) family protein	2.13	1.45	NA
A2G07_RS10460	DR_0603	NUDIX domain-containing protein	1.66	1.50	NA
A2G07_RS10465	DR_0602	phosphoglycerate mutase family protein	1.70	1.70	NA
A2G07_RS11560	DR_0393	elongation factor G	2.40	1.69	NA
A2G07_RS11610	DR_0383	DUF4384 domain-containing protein	2.71	3.31	NA
A2G07_RS14280	DR_A0162	YeeE/YedE family protein, sulphur transport	1.75	1.59	NA
A2G07_RS00965	DR_2524	50S ribosomal protein L28	−1.68	−2.09	NA
A2G07_RS01745	DR_2366	50S ribosomal protein L32	−1.43	−2.60	NA
A2G07_RS04590	DR_1799	translation initiation factor IF-2	−1.49	−2.53	NA
A2G07_RS04595	DR_1798	YlxR family protein	−1.69	−2.21	NA
A2G07_RS04600	DR_1797	transcription termination factor NusA	−1.64	−2.66	NA
A2G07_RS06430	DR_1424	molecular chaperone DnaJ	−1.48	−1.77	NA
A2G07_RS12655	DR_0173	peptide chain release factor 2	−1.60	−1.36	NA
A2G07_RS13065	DR_0086	50S ribosomal protein L21	−1.60	−2.69	NA
A2G07_RS13185	DR_A0304	MBL fold metallo-hydrolase	−7.21	−3.88	NA
A2G07_RS14880	DR_A0290	ATP-dependent zinc metalloprotease FtsH	−2.25	−1.94	NA
A2G07_RS11815	DR_0341	30S ribosomal protein S15	−1.53	−2.87	NA
A2G07_RS03095	DR_2102	type II toxin-antitoxin system VapC family toxin	−1.71	−2.47	NA
A2G07_RS03080	DR_2105	hypothetical protein	−1.55	−2.21	NA
A2G07_RS10160	DR_0661	ribbon-helix-helix domain-containing protein	−1.70	−2.20	NA
A2G07_RS02860	DR_2149	membrane protein insertase YidC	−1.57	−1.93	NA
**Genes with anomalous regulatory patterns**					
A2G07_RS11565	DR_0392	BON domain-containing protein	1.45	NA	−1.38
A2G07_RS14275	DR_A0161	phosphate signaling complex protein PhoU	2.09	NA	−1.44
A2G07_RS08505	DR_0997	Crp-like helix-turn-helix domain-containing protein	NA	−1.76	3.42
**Selected genes that are up/down-regulated simultaneously in both *△J-like* and *△J/△J-like*, but *△J* strain**					
A2G07_RS09370	DR_0826	bifunctional oligoribonuclease, RecJ-like	−4.86	NA	−6.90
A2G07_RS15420	DR_B0125	iron-siderophore ABC transporter substrate-binding protein	−1.91	NA	−3.08
A2G07_RS04845	DR_1749	peptidoglycan endopeptidase LysM	−1.52	NA	−2.62
A2G07_RS13825	DR_A0068	CoA transferase subunit A	−1.67	NA	−1.90
A2G07_RS04020	DR_1917	GNAT family N-acetyltransferase	−2.29	NA	−1.87
A2G07_RS15425	DR_B0124	SIP domain-containing protein	−1.46	NA	−1.85
A2G07_RS11085	DR_0485	glutamate–tRNA ligase	−1.66	NA	−1.65
A2G07_RS07315	DR_1240	CoA ester lyase	−1.53	NA	−1.57
A2G07_RS12780	DR_0148	valine–tRNA ligase	−1.53	NA	−1.53
A2G07_RS14760	DR_A0262	branched-chain amino acid ABC transporter permease	−1.86	NA	−1.52
A2G07_RS02995	DR_2121	branched-chain amino acid ABC transporter permease	−1.50	NA	−1.43
A2G07_RS14755	DR_A0261	branched-chain amino acid ABC transporter ATP-binding protein/permease	−1.88	NA	−1.43
A2G07_RS14725	DR_A0255	N-acyl homoserine lactone acylase QqaR	−1.58	NA	−1.42
A2G07_RS01380	DR_2440	tRNA-dihydrouridine synthase family protein	1.58	NA	1.60
A2G07_RS15955	DR_C0012	LuxR C-terminal-related transcriptional regulator	2.43	NA	1.73
A2G07_RS09365	DR_0827	HAD family phosphatase	1.89	NA	1.83
A2G07_RS11775	DR_0349	endopeptidase La	1.48	NA	1.88
A2G07_RS07940	DR_1114	Hsp20/alpha crystallin family protein	1.96	NA	1.96
A2G07_RS02950	DR_2130	DUF3656 domain-containing protein	1.84	NA	1.97
A2G07_RS00040	DR_0070	single-stranded DNA-binding protein DdrB	2.56	NA	2.04
A2G07_RS12860	DR_0129	molecular chaperone DnaK	1.98	NA	2.27
A2G07_RS12875	DR_0126	DnaJ domain-containing protein	2.40	NA	2.28
A2G07_RS12550	DR_0194	zinc metallopeptidase	1.85	NA	2.34
A2G07_RS08285	DR_1046	ATP-dependent chaperone ClpB	2.70	NA	2.45
A2G07_RS12810	DR_0139	ribosome-binding GTPase HflX	1.45	NA	2.70
A2G07_RS01115	DR_2494	DNA-directed RNA polymerase subunit omega	3.41	NA	2.82
A2G07_RS11055	DR_0492	putative quinol monooxygenase	1.61	NA	2.91
A2G07_RS12865	DR_0128	nucleotide exchange factor GrpE	2.57	NA	2.97
A2G07_RS16310	DR_1813	LiaF-related protein; Cell wall-active antibiotics response 4TMS YvqF	1.49	NA	3.30
A2G07_RS16070	DR_0051	iron-sulfur cluster assembly accessory protein	1.56	NA	3.31
A2G07_RS11060	DR_0491	type 1 glutamine amidotransferase domain-containing protein	1.87	NA	3.34
A2G07_RS06200	DR_1473	PspA/IM30 family protein	2.14	NA	4.14
A2G07_RS15960	DR_C0013	*N*-acetylmuramoyl-l-alanine amidase	6.56	NA	4.20
**Selected genes that are up/down-regulated simultaneously in both *△J* and *△J/△J-like*, but *△J-like* strain**					
A2G07_RS07875	DR_1126	ssDNA-specific exonuclease RecJ	NA	−4.36	−4.10
A2G07_RS15860	DR_C0034	radical SAM protein	NA	−3.62	−2.08
A2G07_RS15500	DR_B0109	ribonucleotide-diphosphate reductase subunit beta	NA	−3.53	−2.41
A2G07_RS15875	DR_C0037	carbamoyltransferase	NA	−3.32	−2.22
A2G07_RS12990	DR_0102	50S ribosomal protein L9	NA	−3.21	−1.38
A2G07_RS17860	DR_0907	DNA damage response modulator PprM	NA	−3.11	−1.60
A2G07_RS02955	DR_2129	50S ribosomal protein L17	NA	−3.07	−1.78
A2G07_RS15505	DR_B0108	class 1b ribonucleoside-diphosphate reductase subunit alpha	NA	−2.93	−2.12
A2G07_RS06015	DR_1510	ribosome recycling factor	NA	−2.75	−1.64
A2G07_RS01225	DR_2472	CarD family transcriptional regulator	NA	−2.52	−2.93
A2G07_RS10315	DR_0631	cell division protein FtsZ	NA	−2.34	−2.07
A2G07_RS07695	DR_1162	transcription elongation factor GreA	NA	−2.28	−1.68
A2G07_RS07185	DR_1266	proline–tRNA ligase	NA	−2.22	−1.93
A2G07_RS10320	DR_0630	cell division protein FtsA	NA	−2.17	−1.79
A2G07_RS07165	DR_1270	asparagine–tRNA ligase	NA	−1.96	−1.82
A2G07_RS01025	DR_2512	RidA family protein, Endoribonuclease L-PSP	NA	−1.96	−1.75
A2G07_RS06795	DR_1347	aspartate–tRNA ligase	NA	−1.89	−2.03
A2G07_RS03195	DR_2081	threonine–tRNA ligase	NA	−1.88	−1.77
A2G07_RS00390	DR_0001	DNA polymerase III subunit beta	NA	−1.83	−1.38
A2G07_RS07130	DR_1276	serine–tRNA ligase	NA	−1.74	−2.02
A2G07_RS06530	DR_1402	TetR/AcrR family transcriptional regulator	NA	−1.56	−2.23
A2G07_RS11665	DR_0372	lysine–tRNA ligase	NA	−1.50	−1.69
A2G07_RS05645	DR_1585	ribonuclease PH	NA	−1.43	−1.44
A2G07_RS04835	DR_1751	uracil-DNA glycosylase	NA	1.81	1.46
A2G07_RS01700	DR_2375	GreA/GreB family elongation factor	NA	1.83	1.52
A2G07_RS00065	DR_0065	DEAD/DEAH box helicase	NA	1.77	1.59
A2G07_RS13950	DR_A0094	terminase family protein	NA	2.22	1.61
A2G07_RS14230	DR_A0152	IclR family transcriptional regulator	NA	1.83	1.65
A2G07_RS12905	DR_0120	DNA-processing protein DprA	NA	1.85	1.66
A2G07_RS00780	DR_2562	class I SAM-dependent methyltransferase	NA	1.75	1.68
A2G07_RS18155	DR_2427	RNA 2′-phosphotransferase	NA	2.04	1.78
A2G07_RS00990	DR_2519	MerR family transcriptional regulator	NA	2.08	1.81
A2G07_RS02640	DR_t40	tRNA-Pro	NA	2.06	2.65
A2G07_RS10845	DR_0530	Bifunctional DNA primase/polymerase, N-terminal	NA	2.09	3.27

^a^Gene ID from RNA seq results.

^b^Gene ID from KEGG annotation.

*log_2_FoldChange.

While among the upregulated and downregulated genes, 37 and 18 genes, respectively, are shared between both the *ΔJ-like* and *ΔJ/ΔJ-like* strains but absent in the *ΔJ* strain (Figure 2B). These genes that are only influenced by DrRecJ-like include the N-acetyltransferase (DR_1917), the peptidoglycan endopeptidase (DR_1749), Siderophore-interacting protein (DR_B0124), ABC transporters related proteins (DR_B0125, DR_2121, DR_A0261, DR_A0262), and *N*-acetylmuramoyl-l-alanine amidase (DR_C0013) (Table 1; Supplementary Table S3), which are related to the cell wall or the cell membrane synthesis or transportation, directly or indirectly. That might explain the phenotype that strains lacking RecJ-like were more sensitive to tween 20. Moreover, the expression of genes encoding protease, peptidase, heat shock proteins, protein chaperones, and assembly accessory proteins (DR_0827, DR_0349, DR_1114, DR_2130, DR_0129, DR_0126, DR_0194, DR_1046, DR_0051, DR_0491 and DR_0128) is also influenced by DrRecJ-like, indicating that the deletion of DrRecJ-like might affect the folding and stability of proteins.

Furthermore, DrRecJ and DrRecJ-like may exert a mutually inhibitory effect on specific pathways. The *△J* strain exhibits 217 unique upregulated genes and 160 unique downregulated genes, while the *△J-like* strain shows 91 unique upregulated genes and 15 unique downregulated genes (Figure 2B). The expression levels of these genes in the double mutant strain did not show significant changes. Interestingly, for certain genes, the expression differences induced by the double mutation are even smaller than those caused by the respective single mutations. For example, there are 9 upregulated genes shared by both *△J* and *△J-like* single mutant strain, which are not upregulated in the *△J/△J-like* double deletion mutant strain (Figure 2B; Table 1; Supplementary Table S3). Additionally, 15 downregulated genes are shared by both *△J* and *△J-like* single mutant strain, but they are not downregulated in the double deletion mutant strain (Figure 2B; Table 1; Supplementary Table S3). The KEGG pathway analysis revealed that among the 15 downregulated genes in the single rather than double deletion mutant strain, seven genes are associated with translation processes (DR_2524, DR_2366, DR_0086, DR_0341 are ribosomal proteins, DR_1799 is the translation initiation factor IF-2, DR_1797 is the transcription termination factor NusA, and DR_0173 is the peptide chain release factor 2), one is related to protein folding (DR_1424, chaperone protein DnaJ), one is involved in cell division (DR_A0290, cell division protein FtsH), and one is related to cellular metabolism (DR_A0304, pyruvate metabolism related protein) (Table 1; Supplementary Table S3). That may help explain why the double deletion mutant strain grows better than either of the single mutants at 37°C.

Additionally, a total of 69 upregulated and 52 downregulated genes showed significant changes in the double mutant strain, whereas their expression changes were not obvious in either of the single mutants (Figure 2B; Supplementary Table S3).

In summary, the RNA-seq results suggest a complex interplay between DrRecJ and DrRecJ-like *in vivo*, involving both overlapping and antagonistic functions.

### DrRecJ-like hydrolyzes several substrates *in vitro*, displaying a strong preference for linear dinucleotides and trinucleotides

**Figure 3.**
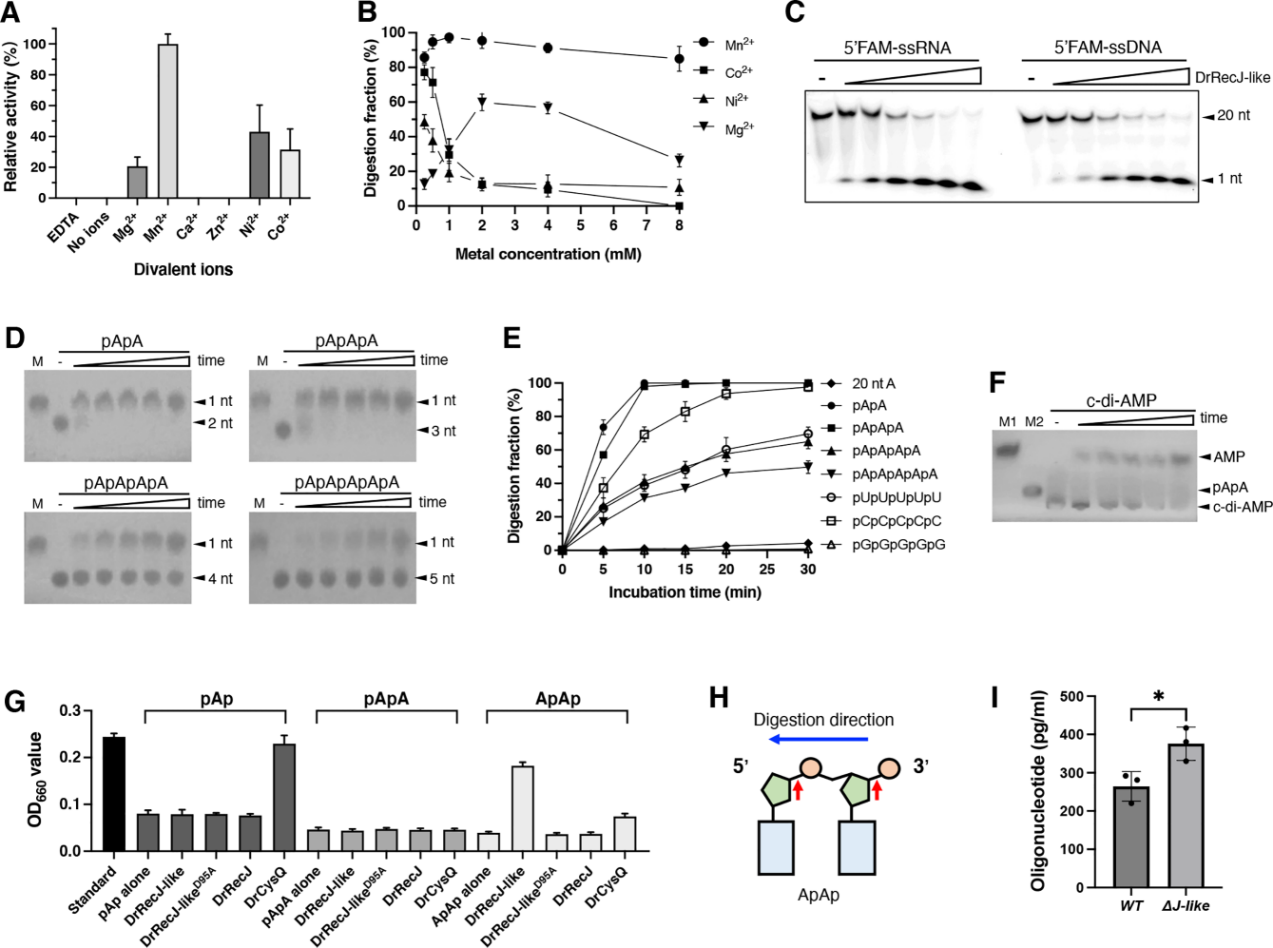
The enzymatic activity analysis of DrRecJ-like. The enzymatic activity analysis of DrRecJ-like. (**A**) Metal preference test. Hydrolysis of 100 nM 20 nt 5′FAM labeled RNA by DrRecJ-like (500 nM) in the presence of indicated divalent metal cations (10 mM). (**B**) Divalent metal ion concentrations test. 100 nM 20 nt 5′FAM labeled ssRNA was incubated with 100 nM DrRecJ-like (when MnCl_2_ or CoCl_2_ was used), or 200 nM DrRecJ-like (when NiCl_2_ was used), or 500 nM DrRecJ-like (when MgCl_2_ was used). Different concentrations (0, 0.25, 0.5, 1.0, 2.0, 4.0 and 8.0 mM) of divalent metal cations were introduced. (**C**) Digestion comparison between ssRNA and ssDNA by DrRecJ-like. 100 nM 20 nt 3′FAM labeled ssRNA or ssDNA was incubated with different concentrations of DrRecJ-like (0, 6.25, 12.5, 25, 50, 100, 200 nM) and 1 mM Mn^2+^. (**D**) Optimum substrate lengths determination. 0.8 mM substrates with different length (2, 3, 4, 5 nt) were incubated with 0.5 μM DrRecJ-like and 1 mM Mn^2+^ for various durations (0, 5, 10, 15, 20, 30 min), and the reaction products were resolved by TLC. M represents AMP standard. (**E**) Comparison of the cleavage efficiency of DrRecJ-like on different substrates based on the TLC results. The reaction conditions are the same as that in figure 3D and Supplementary Figure S3A. Three technical replicates were conducted. (**F**) Digestion assays of c-di-AMP. 0.8 mM c-di-AMP was incubated with 2 μM DrRecJ-like and 1 mM Mn^2+^ for various durations (0, 5, 10, 15, 20, 30 min), and the reaction products were resolved by TLC. M1 represents AMP standard, and M2 represents pApA standard. (**G**) The inorganic phosphate assays testing the digestion of pAp, pApA and ApAp. 200 μM pAp, pApA, or ApAp was incubated with 200 μM DrRecJ-like (or DrRecJ-like^D95A^, or DrRecJ) or 2 μM DrCysQ, and 1 mM Mn^2+^ for 1 h. The release of Pi was detected at OD_660_, by an inorganic phosphate assay kit. 200 μM Pi standard was set as a control. Three technical replicates were conducted. (**H**) A model for catalysis of ApAp by DrRecJ-like. Red arrows indicate the cleavage sites, and the blue arrow indicate the digestion direction. (**I**) The levels of *in vivo* oligonucleotide content were measured in the wild-type and DrRecJ-like deletion strain. The concentrations of oligonucleotides were calculated per milliliter of culture (OD_600_= 1). Three technical replicates were conducted. ‘*’ indicated *P*< 0.05.

To investigate the activity of DrRecJ-like, the non-tagged DrRecJ-like protein was purified. DrRecJ-like was then incubated with a 20 nt ssRNA in reaction buffers containing various metal cations. The results revealed that DrRecJ-like efficiently hydrolyzes ssRNA to 1 nt, with the highest activity observed in the presence of Mn^2+^ as a cofactor (Figure 3A). This observation aligns with findings for homologs from other bacteria (12,14,38–40). Additionally, Ni^2+^, Co^2+^ and Mg^2+^ proved to be effective metal cofactors, while Zn^2+^ and Ca^2+^ exhibited inhibitory effects.

Further exploration involved determining the optimal concentrations of these catalytic metal ions using the same substrate. The results indicated that the optimum concentrations for Mn^2+^, Ni^2+^, Co^2+^ and Mg^2+^ are 1.0, 0.25, 0.25 and 2.0 mM, respectively (Figure 3B).

The phenotype and RNA-seq results suggest that DrRecJ and DrRecJ-like share some functional similarities *in vivo*. Previous reports have indicated that DrRecJ specifically cleaves ssDNA but not ssRNA. Therefore, it is worth investigating whether DrRecJ-like possesses the ability to cleave ssDNA. We conducted a comparative analysis to assess the efficiency of DrRecJ-like in cleaving ssDNA and ssRNA of the same length simultaneously. The results demonstrated that DrRecJ-like exhibits strong cleavage activity towards both types of substrates, with slightly higher activity observed towards ssRNA (Figure 3C). On the contrary, DrRecJ has been reported to lack the ability to cleave RNA due to the spatial hindrance caused by the special structure of its active center towards the 2′-OH of the RNA ribose ring (27,28).

RecJ-like proteins in other species are primarily characterized as nanoRNases due to their affinity for recognizing and cleaving short RNA chains. To determine the optimal substrate length for DrRecJ-like, a series of assays were conducted. TLC was utilized to evaluate the efficiency of DrRecJ-like in cleaving poly A substrates with the same concentration but different lengths (2–5 nt) at various reaction times. The corresponding line graph was generated by comparing substrate degradation and the efficiency of generating 1 nt products at different time points (Figure 3D). The outcomes revealed that DrRecJ-like exhibits the highest cleavage efficiency towards substrates of 2 nt and 3 nt in length. However, as the length increases to 4 nt and 5 nt, the degradation efficiency diminishes rapidly (Figure 3D and E). As anticipated, RecJ-like demonstrated significantly weaker activity (approximately a 100-fold reduction compared to 2–3 nt substrates) towards 20-nt poly A under the same reaction conditions (Figure 3E and Supplementary Figure S3A).

Further analysis of the optimal substrate sequences of DrRecJ-like involved comparing the degradation efficiency of 5-nt poly A, U, C, or G. The results unveiled that the digestion preference of DrRecJ-like follows the order of C > U > A > G (Figure 3E and Supplementary Figure S3A), indicating a slight preference for pyrimidine residues.

We were also interested in determining whether DrRecJ-like can degrade c-di-AMP. To directly assess the phosphodiesterase activity of DrRecJ-like toward c-di-AMP, we incubated the protein with c-di-AMP under different conditions and analyzed the products using TLC. Our results demonstrated that DrRecJ-like efficiently hydrolyzed c-di-AMP into 5′-AMP, with no intermediate product pApA observed (Figure 3F). In contrast, DrRecJ showed no ability to hydrolyze c-di-AMP (Supplementary Figure S3B). Despite using a higher concentration of protein, the product formed by cleaving c-di-AMP was still less than the product formed by cleaving pApA, indicating that DrRecJ-like exhibits a preference for cleaving pApA over c-di-AMP (Figure 3D; Figure 3F).

Subsequently, we employed an inorganic phosphate assay kit to assess the ability of DrRecJ-like to degrade pAp, as TLC may present challenges in distinguishing potential product AMP from substrate pAp. This kit operates on the principle that when the molybdenum blue reagent reacts with phosphate ions (Pi), it forms a substance with a characteristic absorption peak at 660 nm. If the protein can hydrolyze pAp into AMP and Pi, it will result in a change in the OD_660_ value due to its reaction with the molybdenum blue reagent. DrCysQ, expected to hydrolyze pAp into AMP and Pi, served as a positive control. DrRecJ and a putative enzymatically inactive mutant of DrRecJ-like (DrRecJ-like^D95A^, mentioned below) were used as negative controls. It was observed that, in comparison to DrCysQ, even when using DrRecJ-like protein at a concentration 100 times that of DrCysQ, DrRecJ-like demonstrated no ability to hydrolyze pAp into AMP and Pi (Figure 3G). Additionally, neither DrRecJ nor DrRecJ-like^D95A^ exhibited the ability to hydrolyze pAp (Figure 3G). Inorganic phosphate assays were also conducted using pApA or ApAp as substrates. The detection of inorganic phosphate occurred when ApAp was used as the substrate, rather than pApA. This finding suggested that DrRecJ-like digests the substrate to generate 3′OH and 5′P, as opposed to 3′P and 5′OH (Figure 3H), aligning with the digestion mechanism of BsNrnA as elucidated by Schmier *et al.* (41). This assay also helped us confirm the digestion direction of DrRecJ-like, determining whether ApAp is digested into ApA and 5′ P first or A and pAp first. Due to the inability to digest pAp, it is less likely that DrRecJ-like digests ApAp to generate A and pAp first, followed by the digestion of pAp into pA and 5′P. Therefore, the detected inorganic phosphate is more likely derived from the product ApA and 5′ P, suggesting that DrRecJ-like digests the substrate in a 3′-5′ direction (Figure 3H).

We further quantified the accumulation of small oligos in wild-type and DrRecJ-like deletion strains, revealing concentrations of approximately 264 and 376 pg/ml liquid culture (OD_600_= 1.0), respectively, using an oligonucleotide ELISA assay kit (Figure 3I). These results strongly suggest that one of the biological functions of DrRecJ-like is the removal of oligonucleotides.

The outcomes of the enzymatic cleavage experiments mentioned above suggest that DrRecJ-like functions as a canonical NrnA protein, capable of processing both RNA and DNA. It exhibits a distinct preference for shorter oligonucleotides, particularly dinucleotides and trinucleotides, a preference for pyrimidine residues, and acts in a 3′-5′ direction. Additionally, DrRecJ-like has the ability to directly hydrolyze c-di-AMP to form AMPs.

### The structure analysis of DrRecJ-like

**Figure 4.**
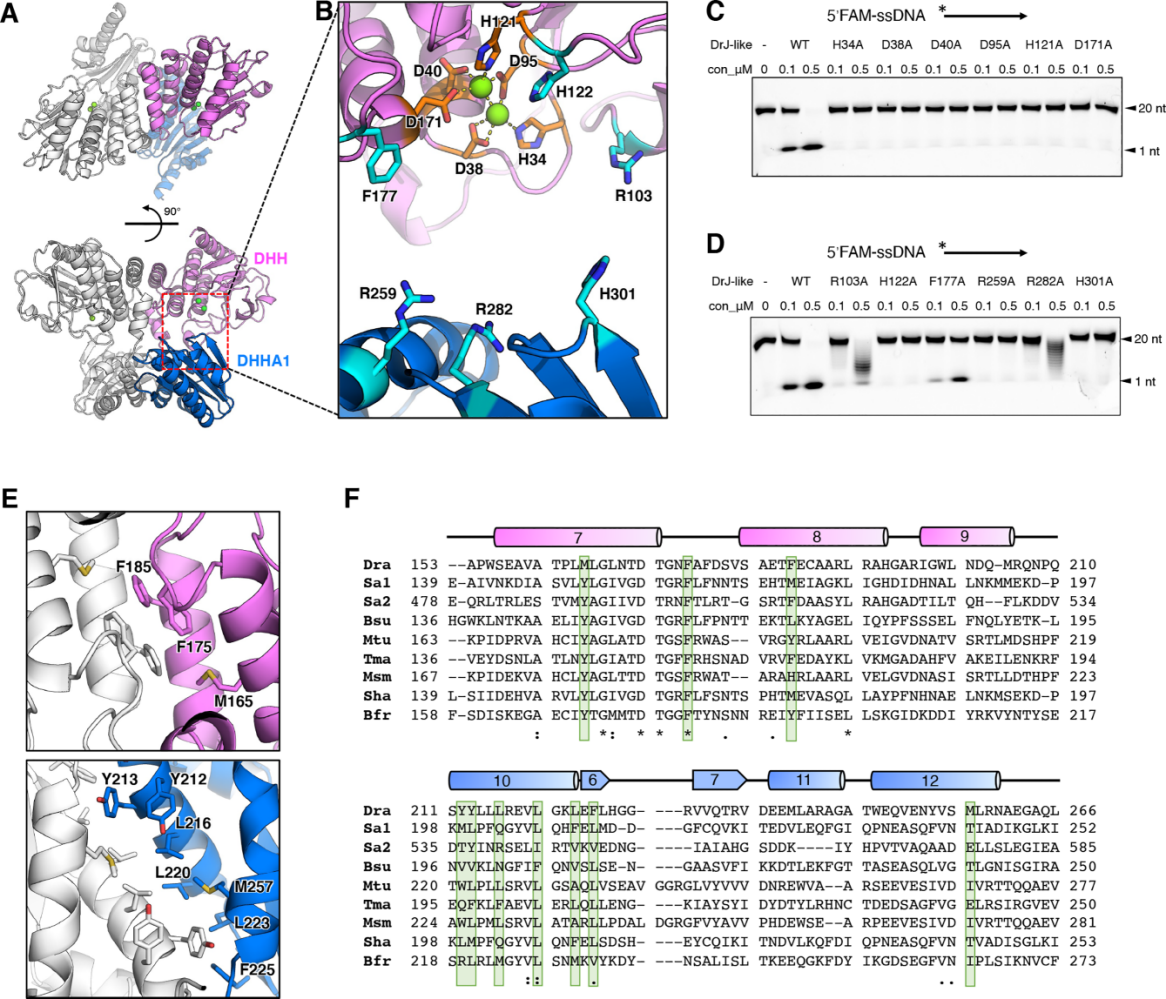
The crystal structure and key residues for catalysis and dimerization of DrRecJ-like. (**A**) The overall structure of DrRecJ-like dimer. One protomer of the dimer is shown in color (DHH domain, violet; DHHA1, marine) and the other protomer of the dimer is shown in white. (**B**) Zoom-in view of the catalytic core. The key residues involved in metal ion coordination and substrate interaction are shown as sticks and colored orange and cyan, respectively. (**C**) and (**D**) Denaturing PAGE gel showing the reduced nuclease activity of point-mutated DrRecJ-like proteins (alanine substitutions of key residues involved in metal ion coordination or substrates binding). 5′FAM-labeled 20 nt ssDNA (200 nM) was incubated with different concentrations of DrRecJ-like proteins (0, 0.1 and 0.5 μM) in the presence of 1 mM Mn^2+^ (see methods). (**E**) Zoom-in view of the dimerization interface. Key residues for dimerization are shown as sticks and labeled. (**F**) Sequence alignment of the dimerization areas of the structured NrnA homologs. Dra, *D. radiodurans* RecJ-like (PDB ID: 8IOO); Sa1, *S. aureus* SA0013 NrnA (PDB ID: 8IU7); Sa2, *S. aureus* SA0013 (GdpP) (PDB ID: 5XSI); Bsu, *B. subtilis* YtqI (PDB ID: 5J21); Mtu, *Mycobacterium tuberculosis* Rv2837c (CnpB) (PDB ID: 5JJU); Tma, *T. maritima* TM1595 (PDB ID: 5O1U); Msm, *Mycobacterium smegmatis* MC2 155 NrnA (PDB ID: 4LS9); Sha, *Staphylococcus haemolyticus* JCSC1435 NrnA (PDB ID: 3DEV); Bfr, *Bacteroides fragilis* YCH46 (PDB ID: 3W5W). Secondary structural elements are depicted according to the structure of the DrRecJ-like protein characterized in this study and displayed at the top of sequences. Putative residues involved in protein dimerization are boxed in light green.

The full-length DrRecJ-like protein, free from any substrate or product, was successfully crystallized in space group C121. The structure was solved at a resolution of 1.97 Å using the molecular replacement method with the apo structure of *Thermotoga maritima* PDE (PDB code: 5O1U) as the search model (Figure 4A). Detailed crystallographic statistics are presented in Table 2. The refined DrRecJ-like structure comprises three protomers in the asymmetric unit, with two of the protomers forming a dimer and the remaining protomer forming a dimer with a symmetrical protomer from a neighboring molecule. The three protomers can be superimposed well, with the root-mean-square deviation (RMSD) values between each protomer <1.0 Å (Supplementary Figure S4A). Further superimposition of DrRecJ-like with the substrate-bound *M. tuberculosis* Rv2837c structure (MtNrnA, PDB ID: 5JJU) indicated that the present DrRecJ-like structure is in an open state, with poorly occupied substrate-binding sites (Supplementary Figure S4B).

**Table 2.** Statistics from crystallographic analysis

Complex	DrRecJ-like
PDB code	8IOO
**Data collection**	
Source	BL02U1
Wavelength (Å)	0.9790
Resolution (Å)	96.67–1.97 (2.07–1.97)
Space group	*C*121
Cell dimensions: a, b, c α, β, γ	152.29, 124.84, 80.917 90, 92.716, 90
Observation	308 794 (15 414)
Unique reflections	106 336 (7618)
*R*_merge_ (%)	5.4 (35.8)
*I*/σ*I*	8.7 (1.7)
Completeness (%)	99.5 (96.3)
Redundancy	2.9
**Refinement statistics**	
Resolution (Å)	96.67–1.97 (2.07–1.97)
*R*_factor_ (%)/R_free_ (%)	22.76/24.38
rmsd bonds (Å)/angles (°)	0.010/1.245
Ramachandran plot: favored (%)/outliers (%)	96.5/0

The numbers in parentheses refer to the outer shell.

*R*_factor_ = Σ||*F*(obs) – *F*(calc)||/Σ|*F*(obs)|.

*R*_free_ = *R* factor calculated using 5.0% of the reflection data randomly chosen and omitted from the start of refinement.

Although our crystals were soaked with the putative product, AMP, before being frozen, there was no AMP density in the map. This suggests that the open state of the DrRecJ-like protein has very weak affinity for its cleavage products. The crystallization condition used in our study contained MgSO_4_, and we observed two Mg^2+^ ions bound at the active site. SO_4_^2−^ groups are highly acidic and are commonly used as mimics of PO_4_^2−^ groups found in nucleotides to predict nucleotide binding surfaces. However, we did not observe any SO_4_^2−^ density near the active site, further suggesting that in this open state, the active site has a weak binding affinity for its nucleic acid products or substrates.

Like other structurally characterized NrnA homologs, each protomer of DrRecJ-like consists of a conserved DHH domain, in which five stranded β-sheets (21345, ↑↑↑↑↑) are sandwiched between a series of α-helices, and a conserved DHHA1 domain, in which six stranded β-sheets (123465, ↓↑↑↓↑↓) are sandwiched between a series of α-helices (Figure 4A; Supplementary Figure S4).

DrRecJ-like shared a conserved DHH catalytic core with reported NrnA structures from other bacteria (Supplementary Figure S5). Two catalytic metal ions (Mg^2+^) coordinated by conserved histidine and aspartate residues (H34, D38, D40, D95, H121, and D171) were observed in the catalytic core (Figure 4B). The second ‘H’ of the DHH motif (H122) was not directly bound to the catalytic metal ions. It may interact with the phosphate group of the substrate and act as a holder to correctly orient the phosphodiester bond for nucleophilic attack, as suggested in the case of the DrRecJ protein (27). Alanine substitutions of these residues caused an almost complete inactivation of DrRecJ-like (Figure 4C and D), consistent with the results of equivalent mutations in DrRecJ (28).

Based on the substrate-bound MtNrnA structure (42), we hypothesized that residues F177 on DrRecJ-like DHH domain may form stack interaction with the base ring of the substrate, while residue R103 on the DHH domain, and residues R259, R282 and H301 on the DHHA2 domain may bind with the phosphate groups of the substrate (Figure 4B). Alanine substitutions of these residues reduced the DrRecJ-like activities (Figure 4D). Due to the high processivity of the wild-type DrRecJ-like protein, only 1-nt products can be observed on the gel for substrates labeled with either 3′ or 5′ FAM (Figure 4C and D; Supplementary Figure S6). Thus, we were unable to determine the directional preference of DrRecJ-like for substrate cleavage. However, mutations at residues R103 and R282 affected the processivity of DrRecJ-like. This resulted in the generation of multiple ladder bands for 5′ FAM-labeled substrates rather than 3′ FAM-labeled substrates (Figure 4D; Supplementary Figure S6). Therefore, we proposed that DrRecJ-like cleaves longer oligos in the 3′-5′ direction, similar to the nanoRNA mentioned above.

All the NrnA studied thus far are in biological dimers, or a dimer of dimers (16). According to the structural data, dimerization of DrRecJ-like is mediated by hydrophobic interactions between several α-helices (the α7 and α8 on the DHH domain, and the α10 and α12 on the DHHA1 domain) of protomers (Figure 4E). We compared the amino acid sequences of the dimerization surfaces of DrRecJ-like and other structurally characterized NrnA homologs, revealing that several conserved hydrophobic amino acids might mediate the dimerization of this protein family (Figure 4F; Supplementary Figure S5).

### Dimerization is essential for the biological function of DrRecJ-like

**Figure 5.**
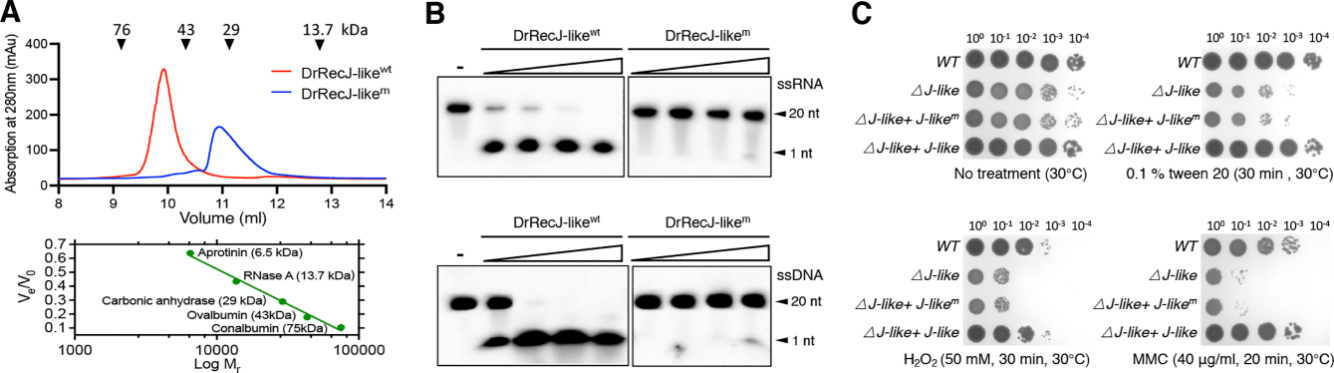
Functional analysis of the dimerization of DrRecJ-like. (**A**) Gel filtration confirmation of the DrRecJ-like dimerization-defective mutant. A set of protein standards with known molecular masses, including aprotinin (6.5 kDa), RNase A (13.7 kDa), carbonic anhydrase (29 kDa), ovalbumin (43 kDa), and conalbumin (75 kDa), were used to calibrate the Superdex 75 10/300GL. DrRecJ-like^wt^ eluted around 10 ml, indicating a dimeric form. DrRecJ-like^m^ eluted around 11 ml, indicating a monomeric form. (**B**) Comparison of the digestion activities of DrRecJ-like^wt^ and DrRecJ-like^m^ towards oligonucleotides. 200 nM 5′FAM-labeled 20 nt ssDNA or ssRNA was incubated with different concentrations of DrRecJ-like proteins (0, 0.1, 0.2, 0.4 and 0.8 μM) in the presence of 1 mM Mn^2+^ (see methods). (**C**) The WT, *△J-like*, and *△J-like* strains complemented with DrRecJ-like^wt^ or DrRecJ-like^m^ (*△J-like + J-like* or *△J-like + J-like^m^*) were treated with 0.1% tween 20 (for 30 min), 50 mM H_2_O_2_ (for 30 min), FUV (for 400 J/m^2^), or 40 μg/ml MMC (for 30 min). Cells were diluted and spotted on plates. Plates were cultured for 2−3 days at 30°C.

To investigate the function and significance of DrRecJ-like dimerization, we generated a DrRecJ-like dimerization-defect mutant, denoted as DrRecJ-like^m^, with three-point mutations (F185A, Y213A, and L220A) on the putative dimerization surface. Analytical gel filtration confirmed the dimerization deficiency of DrRecJ-like^m^; while wild-type DrRecJ-like eluted as a dimer, DrRecJ-like^m^ eluted as a monomer (Figure 5A). Moreover, the highly asymmetrical elution peak indicated that DrRecJ-like^m^ might stay in a flexible form, suggesting that disruption of its dimerization would impact the protein stability.

Compared to wild type DrRecJ-like, DrRecJ-like^m^ exhibited significantly reduced enzymatic activity towards ssRNA (Figure 5B). Moreover, the DrRecJ-like^m^ mutant failed to restore the wild-type phenotype when treated with 0.1% tween 20, H_2_O_2_ or MMC (Figure 5C). Both the enzymatic cleavage and phenotypic experiments confirmed the critical importance of dimerization in the biological function of DrRecJ-like.

## Discussion

In our previous investigations, we observed slowed growth and temperature sensitivity in the DrRecJ-deficient strain, yet a comprehensive explanation has remained elusive until now (27,28). In this study, RNA-seq results revealed transcriptional changes in various replication, transcription, translation, and division-related proteins in the RecJ-deficient strain, providing insights into the aforementioned phenotypes of slowed growth (or halted growth at 37°C) observed in the strain lacking RecJ. Among these, genes encoding DNA polymerase III subunit beta (DR_0001), cell division proteins FtsA (DR_0630) and FtsZ (DR_0631), ribosome proteins (DR_0102 and DR_2129), ribosome recycling factor (DR_1510), transcription elongation factor GreA (DR_1162), and numerous tRNA ligases (DR_1266, DR_1270, DR_1276, DR_1347, DR_2081, and DR_0372) were downregulated.

However, as the DrRecJ protein is a DNA-specific nuclease and does not participate in the degradation of small signaling molecules (such as c-di-A(G)MP, pAp, cA(G)MP), its degradation of DNA into deoxyribonucleotides leaves unanswered questions about how this process influences differences in gene transcription levels. It probably affects the transcriptome through a different pathway, perhaps not by directly influencing intracellular transcription levels or mRNA levels. Moreover, studies of RecJ homologs in other bacteria predominantly highlight their involvement in DNA repair, with limited discussion regarding their contributions to cellular replication and division (43). This observation could imply the unique nature of DrRecJ’s intracellular functionality, given its nonconserved yet essential C-terminus (28,30).

Although homologs of RecJ-like proteins have been demonstrated to be involved in RNA metabolism and the degradation of the second messenger c-di-A(G)MP in numerous bacteria, no studies have been conducted to date on the impact of this protein on bacterial transcriptomes. Our research has revealed, for the first time, that the absence of DrRecJ-like in *D. radiodurans* leads to the upregulation of DNA damage response proteins, protease/peptidase, and protein chaperones under normal 30°C growth conditions, suggesting that DrRecJ-like plays a crucial role in responding to external stress and maintaining intracellular protein stability. Despite DrRecJ-like being further confirmed to digest DNA as DrRecJ does, phenotypic experiments and RNA-seq data indicate that these two proteins have functional overlap in only a few pathways. Interestingly, in specific pathways, these two proteins even exhibit mutual repression, the specific mechanism of which remains to be elucidated.

Our biochemical study characterized DrRecJ-like as an NrnA-like protein that preferentially digests short RNA/DNA oligos and can directly hydrolyze c-di-AMP to 5′-pA via the intermediate 5′-pApA, despite exhibiting no hydrolytic activity towards pAp. Therefore, in the following discussion, we will refer to DrRecJ-like as DrNrnA. Structural characterization and point mutation analysis identified key residues for substrate binding and enzymatic processivity. However, the full structural basis of DrNrnA’s preference for its optimum substrate has not yet been fully resolved. Therefore, it is not yet clear whether specific motifs influence the substrate recognition. It is worth noting that several key amino acids, such as R103 and R259 in DrNrnA, are not completely conserved; they are often non-basic amino acids in other homologs. Whether the corresponding sites of these amino acids are also involved in the recognition and binding of substrates by other NrnA-like proteins has not been biochemically confirmed. Interestingly, studies on the activity of NrnA-like proteins in different bacteria often yield very different conclusions, especially regarding the optimal bases, optimal substrate length and the confirmation of cleavage directionality (44). For example, *B. subtilis* NrnA (BsNrnA) possesses a slight preference for 5′ purine residues (38), while DrNrnA prefers digesting pyrimidine residues. NrnAs from *M. tuberculosis* and *Mycoplasma pneumoniae* M129 are known to preferentially digest ssRNA substrates of 2 and 5 nt in length, respectively (17), while DrNrnA prefers digesting 2–3 nucleotide long RNA. Unlike BsNrnA, which degrades 2–5 nucleotide long RNA oligomers from the 3′ end, and longer RNA substrates from the 5′ end (41), our data indicate that DrNrnA cleaves both nanoRNA and longer oligos in the 3′-5′ direction. On the contrary, it was reported that NrnA-like proteins from *Thermus thermophiles* HB8, *Enterococcus faecalis* and *Streptococcus pyogenes* prefer to digest substrate in a 5′-3′ direction (38,45). Further research is required to test whether those non-conservative amino acid variances mentioned above may result in distinct substrate sequence and length preferences, and cleavage directionality of these proteins.

A significant number of RNA enzymes identified to date operate as dimers (or polymer of dimers), including examples like RNase III, RNase Mini-III, RNase Z, RNase BN, RNase E, RNase G, RNase T, Orn, NrnC, RNase J, RNase Y, YqgF and the NrnA-like proteins discussed in this study. Among them, the substrates of RNase III and RNase Mini-III are dsRNA. The dimerization of RNase III or RNase Mini-III monomers creates a cleft that can accommodate the dsRNA substrate and cleaves on both sides of the double strand (46,47). Interestingly, the substrate structures and sequences for other RNA enzymes above display an absence of symmetry, implying that the requirement for their dimerization goes beyond substrate characteristics. The structure of RNase Z is a dimer of metallo-β-lactamase domains, with one subunit in a conformation that coordinates two zinc ions and the other subunit that does not coordinate zinc and is apparently inactive for catalysis but is required for substrate recognition (48). RNase BN, a member of the RNase Z family, shares a highly similar structure with RNase Z. This suggests that its dimerization likely serves a purpose akin to that of RNase Z (49). From the complex structure of RNase E with ssRNA, one protomer in the dimer primarily engages in RNA binding, while the other is mainly responsible for catalyzing the cleavage (50). It's only through their concerted action that enzymatic hydrolysis is accomplished. RNase G, being a truncated version of RNase E, likely shares a similar dimerization mechanism, akin to RNase E (51). In the dimeric structure of RNase T, the catalytic center on one monomer closely interacts with a large basic patch responsible for substrate binding on the other monomer, thus forming the complete active site (52). This structural arrangement elucidates the necessity for RNase T dimerization to carry out its function. As for Orn and NrnC, dimerization biases them towards diribonucleotides as substrates, as the active site of molecule A, together with the conserved residues on adjacent molecule B, form a specialized pocket of a specific size, preventing the recognition and binding of longer substrates (6,10). The substrates for RNase J, RNase Y and YqgF are exclusively ssRNA. Disruption of their dimerization significantly compromises their catalytic activity (53–55). Nonetheless, the precise underlying mechanism from a structural perspective remains to be elucidated. Regarding NrnA-like proteins, while we have established the essential role of dimerization in their functionality, the specific mechanistic details remain elusive. Structurally, there seems to be no direct correlation between substrate binding and catalysis of the two monomers within the NrnA-like dimer. The linker connecting the DHH and DHHA1 domains is relatively flexible, and the substrate-binding and catalysis are dominated by DHHA1 and DHH, respectively. This implies that precise alignment of both domains is required for the substrate to undergo catalytic cleavage. We hypothesize that dimerization maintains a consistent trajectory between the DHH and DHHA1 domains, thereby enhancing the efficiency of substrate binding/release and enzymatic cleavage.

Interestingly, in addition to RecJ and NrnA-like proteins, many bacteria also possess other members of the DHH/DHHA1 family. For example, pde1 in *S. pneumoniae* D39, GdpP in *S. aureus* (56,57), *S. pyogenes* (58), *Streptococcus suis* serotype 2 (59), and *Streptococcus mutans* (60), all contain extra N-terminal transmembrane domains alongside the DHH/DHHA1 domains. Unlike NrnA, these proteins can exclusively catalyze the breakdown of c-di-A(G)MP to 5′-pA(G)pA(G). NrnB, a second nanoRNase in *B. subtilis*, prefers to digest short RNA (12). Additionally, in M. pneumoniae, PdeM is another stand-alone DHH/DHHA1 member besides NrnA, capable of hydrolyzing c-di-AMP (61). In *D. radiodurans*, the DHH/DHHA1 family is represented by only two proteins: DrRecJ and DrNrnA. While DrRecJ’s involvement in DNA metabolism is well-established, the presence of DrNrnA prompts inquiries into its potential functional overlap with homologs like GdpP, NrnB and PdeM found in other species. This raises the question of whether the bacterium depends on these proteins for functionality, or if alternative functional counterparts are at play.

## Supplementary Material

gkae451_Supplemental_Files

## Data Availability

All data generated or analysed during this study are included in this published article, its supplementary information files and publicly available repositories. The RNA-seq data generated in this study has been submitted to the NCBI Gene Expression Omnibus (GEO) under accession number GSE244345. Atomic coordinate and structure factor for the reported crystal structure have been deposited with the Protein Data bank (http://www.wwpdb.org) under accession number 8IOO (DOI: 10.2210/pdb8ioo/pdb).
