# Supplementary material for: Structural and functional investigation of the DHH/DHHA1 family proteins in Deinococcus radiodurans
Source: Nucleic Acids Res. 2024 May 28;52(12):7142–57. doi: 10.1093/nar/gkae451 (PMC11229311; doi:10.1093/nar/gkae451)
Supplement: gkae451_Supplemental_Files [file gkae451_supplemental_files.zip › 0826_SI_v2.pdf]

Structural and functional investigation of the DHH/DHHA1 family proteins in *Deinococcus radiodurans*

Ying Wang[1]†, Wanshan Hao[1]†, Ziming Guo[1], Yiyang Sun[1], Yu Wu[1], Yukang Sun, Tianwen Gao, Yun Luo[1], Lizan Jin[1], Jieyu Yang[1], Kaiying Cheng[1,2]#

[1] Zhejiang Key Laboratory of Medical Epigenetics, Department of Immunology and Pathogen Biology, School of Basic Medical Sciences, Affiliated Hospital of Hangzhou Normal University, Hangzhou Normal University, Hangzhou, 311121, China.

[2] State Key Laboratory for Diagnosis and Treatment of Infectious Diseases, The First Affiliated Hospital, College of Medicine, Zhejiang University, Hangzhou, 310003, China.

†These authors have contributed equally to this work and share first authorship.

#To whom correspondence should be addressed. E-mail: kaiyingcheng@hznu.edu.cn

**Supplementary table S1. Oligos used in this study.**

**(A)** Primers used for cloning and mutagenesis.

| Primers | Sequence (5′→3′) |
|---|---|
| drRecJ-like_up_F | gcgcatcgaagcgggcaa |
| drRecJ-like_up_R | tttaagcttgtaatcaggggtggcagag |
| drRecJ-like_down_F | tttggatccgcaggcgacggcagagta |
| drRecJ-like_down_R | cgtgaagggcgggcaacg |
| drRecJ-like_P1 | ttaccggcggaacgtcggag |
| drRecJ-like_P2 | gcgccagctcggcttcaatg |
| drRecJ-like_FN | ctgtattttcagggccatatgaccgccgaaaactctgc |
| drRecJ-like_RB | acggagctcgaattcggatccttactctgccgtcgcctg |
| drRecJ-like_H34A_F | gtcggggttctcggccgacagcaccacg |
| drRecJ-like_H34A_R | cgtggtgctgtcggccgagaaccccgac |
| drRecJ-like_D38A_F | gcgcgtcgccggcggggttctcgt |
| drRecJ-like_D38A_R | acgagaaccccgccggcgacgcgc |
| drRecJ-like_D40A_F | gccgagcgcggcgccgtcggg |
| drRecJ-like_D40A_R | cccgacggcgccgcgctcggc |
| drRecJ-like_D95A_F | gttgttgtccacggccagcacggcggc |
| drRecJ-like_D95A_R | gccgccgtgctggccgtggacaacaac |
| drRecJ-like_R103A_F | cgcccgccaccgccacggggtcgt |
| drRecJ-like_R103A_R | acgaccccgtggcggtggcgggcg |
| drRecJ-like_H121A_F | ggtgccgtgggcatccacgttgaccactgg |
| drRecJ-like_H121A_R | ccagtggtcaacgtggatgcccacggcacc |
| drRecJ-like_H122A_F | cgcaggttggtgccggcgtgatccacgttgac |
| drRecJ-like_H122A_R | gtcaacgtggatcacgccggcaccaacctgcg |
| drRecJ-like_F175A_F | ccgagtcgaaggcggcgttgccggtgtcgg |
| drRecJ-like_F175A_R | ccgacaccggcaacgccgccttcgactcgg |
| drRecJ-like_F177A_F | cgctcaccgagtcggcggcgaagttgccgg |

| | |
|---|---|
| drRecJ-like_F177A_R | ccggcaacttcgccgccgactcggtgagcg |
| drRecJ-like_F185A_F | cgccgcacactcggcggtctcggcgctc |
| drRecJ-like_F185A_R | gagcgccgagaccgccgagtgtgcggcg |
| drRecJ-like_Y213A_F | gcgcagcagcagggcgtacgactggggg |
| drRecJ-like_Y213A_R | cccccagtcgtacgccctgctgctgcgc |
| drRecJ-like_L220A_F | aattccagtttgcccgccacctcgcgcagcag |
| drRecJ-like_L220A_R | ctgctgcgcgaggtggcgggcaaactggaatt |
| drRecJ-like_R259A_F | accctcggcgttagctaacatgctcacgtagttttcga |
| drRecJ-like_R259A_R | tcgaaaactacgtgagcatgttagctaacgccgagggt |
| drRecJ-like_R282A_F | cgggccgcgcgaggccagcgagaacttg |
| drRecJ-like_R282A_R | caagttctcgctggcctcgcgcggcccg |
| drRecJ-like_H301A_F | tgcggggacggcaccgccgccgcc |
| drRecJ-like_H301A_R | ggcggcggcggtgccgtccccgca |
| drCysQ_FN | tacttccaaggtcatatgctttacgcccccgaac |
| drCysQ_RB | gagctcgaattcggatcctcacttgaccagtccagcg |

**(B)** Oligos used for enzymatic assays.

| **Name** | **Sequence (5′→3′)** |
|---|---|
| pAp | pAp |
| ApAp | ApAp |
| pApA | pApA |
| pApApA | pApApA |
| pApApApA | pApApApA |
| pApApApApA | pApApApApA |
| pUpUpUpUpU | pUpUpUpUpU |
| pGpGpGpGpG | pGpGpGpGpG |
| pCpCpCpCpC | pCpCpCpCpC |
| 20 nt A | AAAAAAAAAAAAAAAAAAAA (RNA) |
| 5'FAM-ssRNA | FAM-AAAAAAAAAAAAAAAAAAAA (RNA) |
| 5'FAM-ssDNA | FAM-AAAAAAAAAAAAAAAAAAAA (DNA) |
| 3'FAM-ssDNA | AAAAAAAAAAAAAAAAAAAA-FAM (DNA) |

**Table S2 Summary of sequence reads statistics obtained from Illumina deep.**

| **Sample name** | **WT** | *△J* | *△J-like* | *△J/△J-like* |
|---|---|---|---|---|
| **Total reads** | 15576394 | 15027674 | 15214422 | 15477354 |
| Total mapped reads | 15292510 | 14869332 | 14666254 | 15227585 |
| Uniquely mapped reads | 14325957 | 14461862 | 14107068 | 14729914 |
| Clean bases | 2.3 G | 2.3 G | 2.3 G | 2.3 G |
| Error rate (%) | 0.03 | 0.03 | 0.03 | 0.03 |
| Q20 (%) | 97.69 | 97.6 | 97.98 | 97.69 |
| Q30 (%) | 93.65 | 93.45 | 94.26 | 93.59 |
| Total mapping rate (%) | 98.18 | 98.95 | 96.40 | 98.39 |
| Uniquely mapping rate (%) | 91.97 | 96.23 | 92.72 | 95.17 |

WT, wild type strain R1; *ΔJ*, *drrecJ* single deletion mutant; *ΔJ-like*, *drrecJ-like* single deletion mutant; *ΔJ/ΔJ-like, drrecJ* and *drrecJ-like* double deletion mutant.

## Supplementary figures

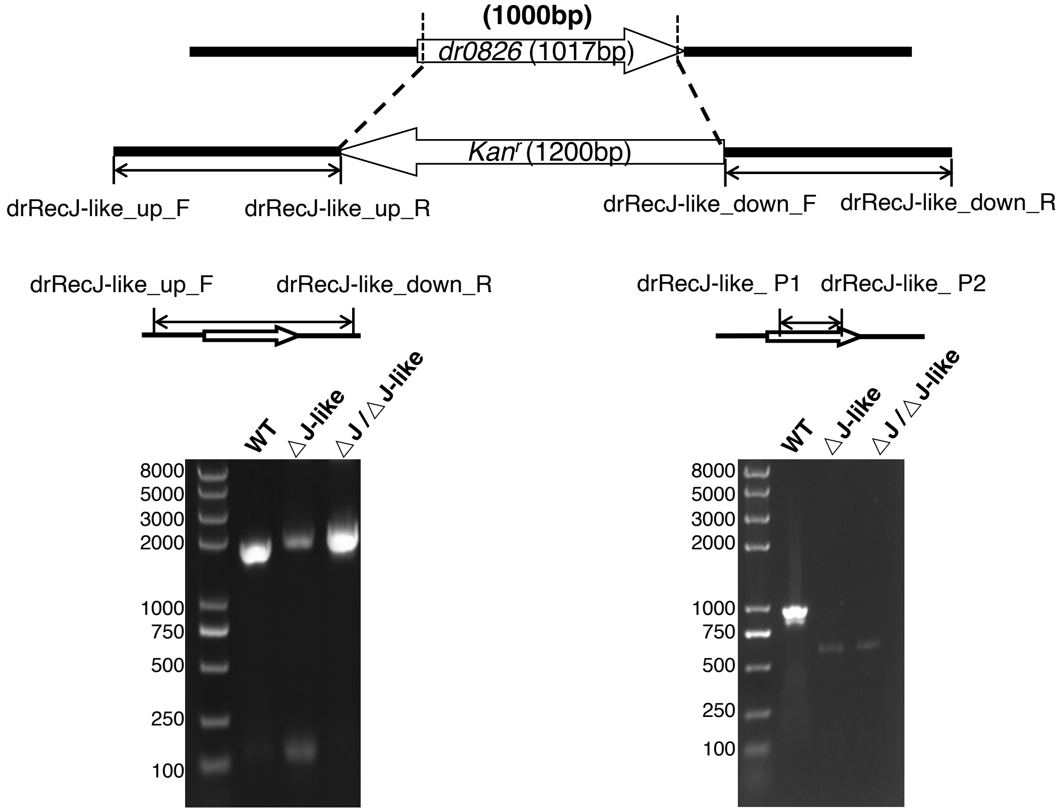


**Supplementary figure S1. Construction and verification of the deletion mutants.** Upper, schematic of the constructions of *drrecJ-like* or *drrecJ/drrecJ-like* deletion mutants. The map of *drrecJ-like* in the *D. radiodurans* chromosome before (top) or after (bottom) replacement with a *kanamycin* resistance cassette are shown. Lower, ethidium bromide-stained agarose gel illustrating that the mutants carry homozygous deletions of *drrecJ-like*. WT, wild type strain; ΔJ-like, *drrecJ-like* single deletion mutant; ΔJ/ΔJ-like, *drrecJ/drrecJ-like* double deletion mutant.

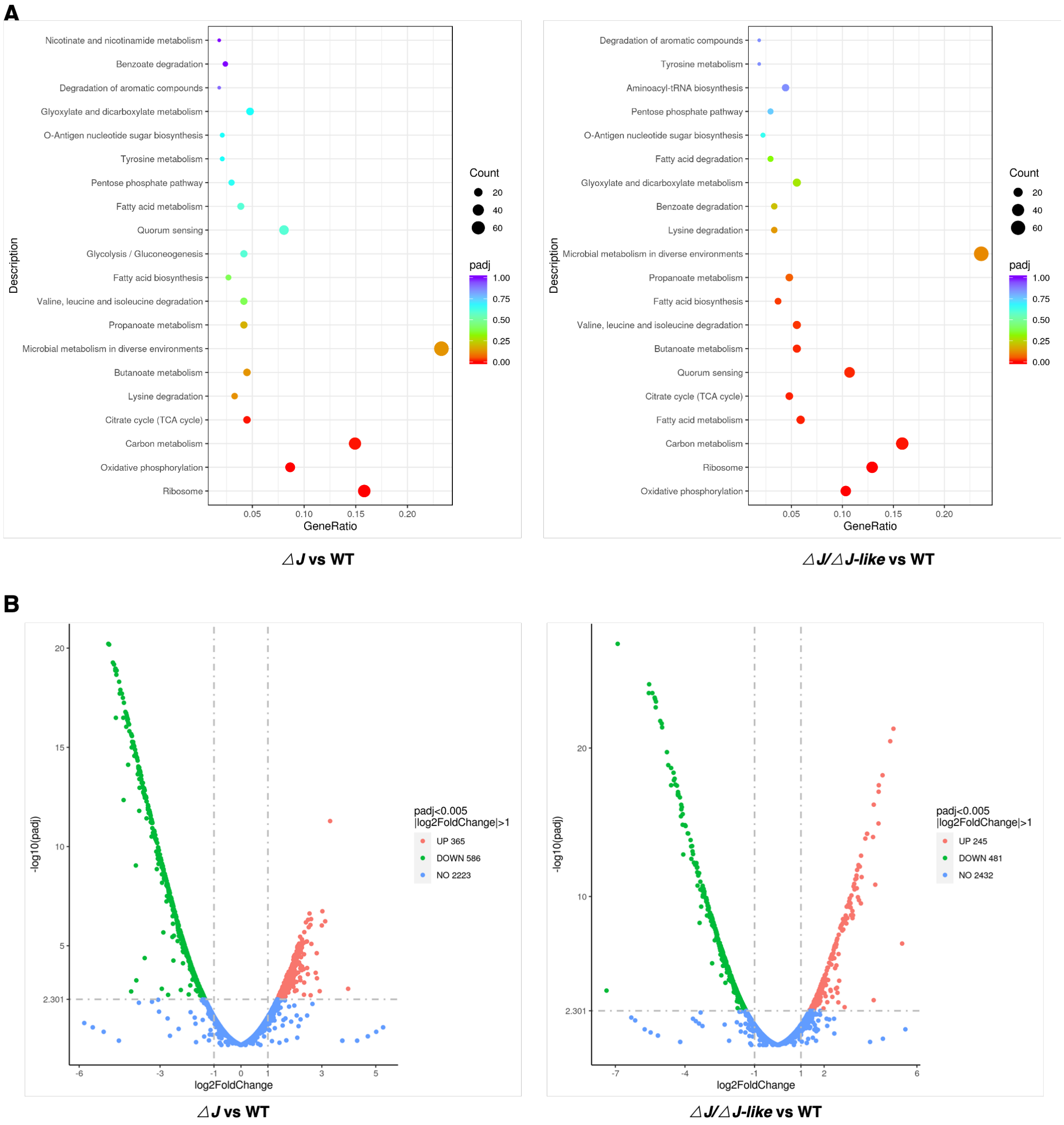


**Supplementary figure S2. The top20 KEGG enrichment pathways and Volcano plot analysis for DEGs.**

(A) The top20 KEGG enrichment pathways for DEGs in pairwise comparison of two groups, *ΔJ* vs WT (left) and *ΔJ /ΔJ-like* vs WT (right). The rich factor refers to the ratio of the number of DEGs in the pathway and the number of all annotated genes in the pathway.

(B) Volcano plot analysis showing DEGs profile between *ΔJ* mutant and WT strain (left), and *ΔJ /ΔJ-like* mutant and WT strain (right). The gray dashed line indicates |log2 fold change|＞1 and adjusted p-value＜0.005.

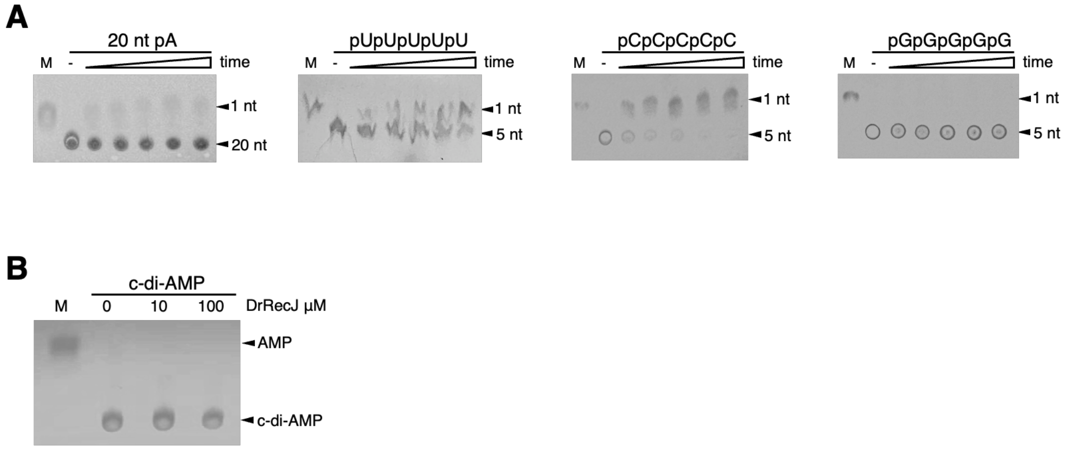


**Supplementary figure S3. The TLC analysis.**

(A) Optimum substrate lengths and optimum substrate sequences determination. The reaction conditions are the same as that in Figure 3D.

(B) C-di-AMP digestion assays of DrRecJ. 0.8 mM c-di-AMP was incubated with 10 or 100 μM DrRecJ and 1 mM $Mn^{2+}$ for 30 minutes, and the reaction products were resolved by TLC. M represents AMP standard.

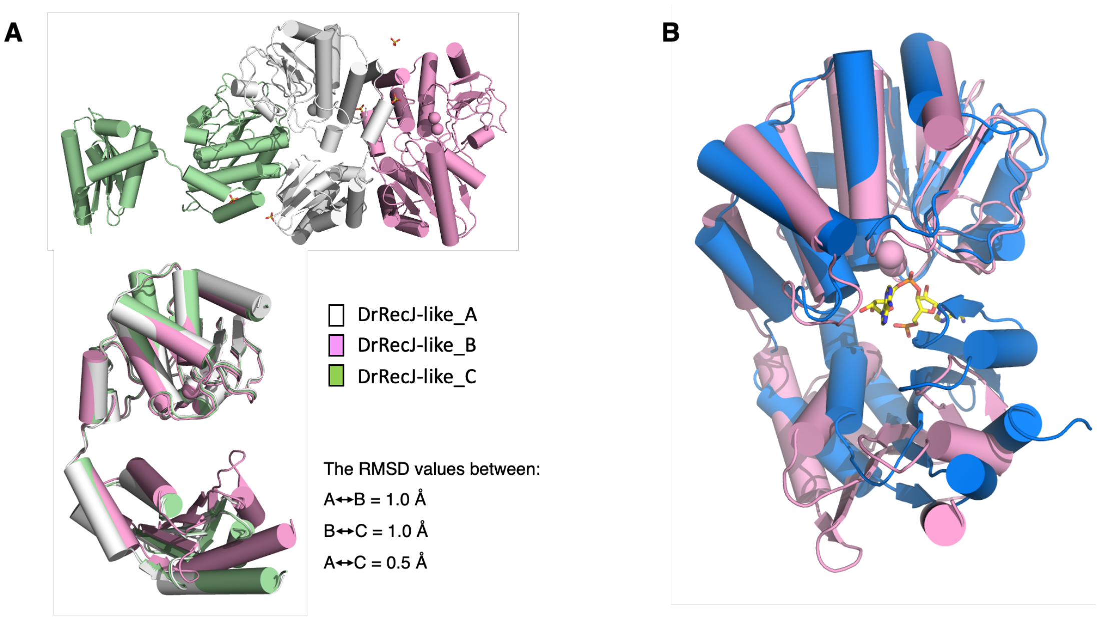


**Supplementary figure S4. Structure superimposition analysis of NrnAs.**

(A) The superimposition of the DrRecJ-like three protomers in the asymmetric unit. Top, the distribution of the DrRecJ-like three protomers in the asymmetric unit. Bottom, The superimposition of protomer A, B and C in the asymmetric unit. Protomer A, B and C were shown in white, pink and palegreen, respectively. $PO_4^{2-}$ groups were shown as sticks. The RMSD values were calculated between each protomer.

(B) The superimposition of DrRecJ-like and the substrate bound MtNrnA (PDB ID: 5JJU). DrRecJ-like and MtNrnA were shown in pink and marine, respectively. The pApA substrate was shown in yellow.

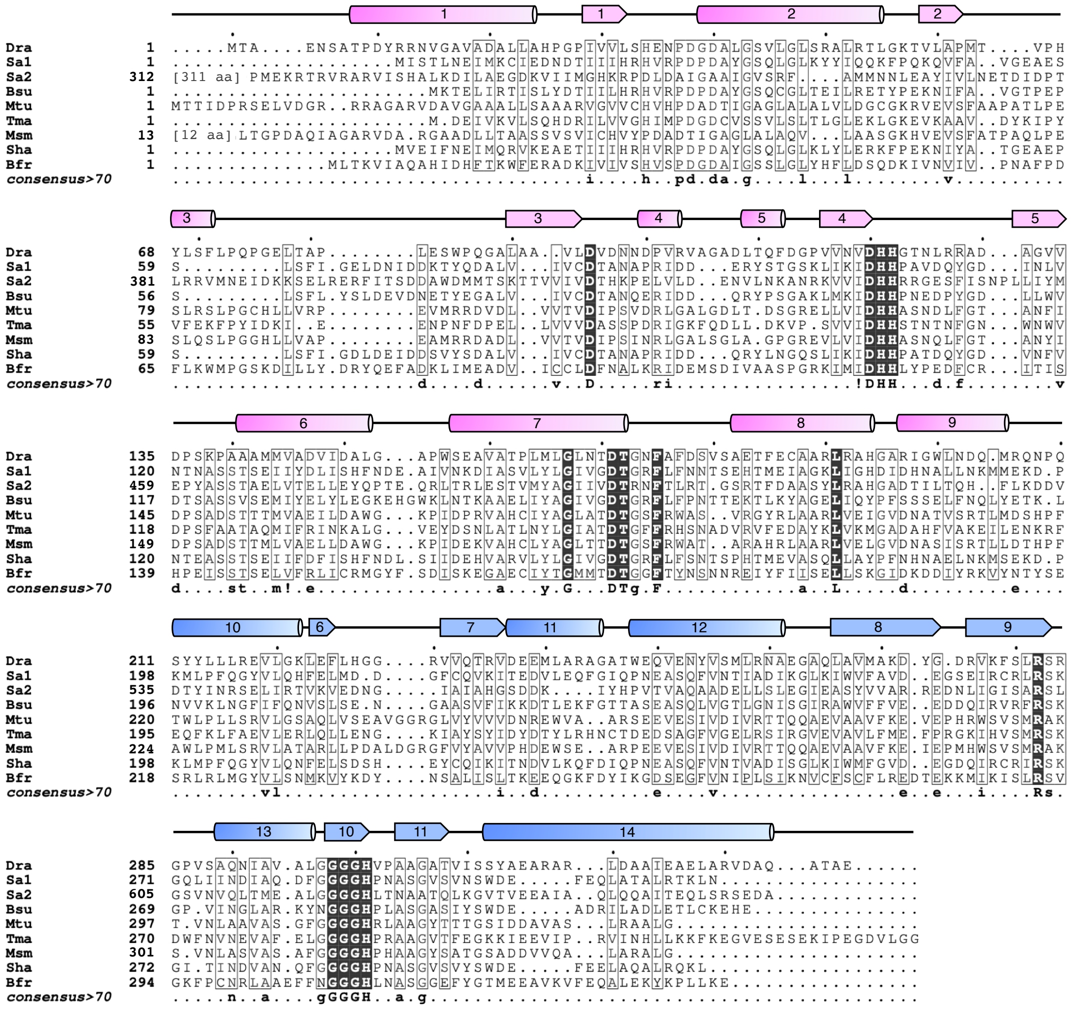


**Supplementary figure S5. Sequence alignments of the structured NrnA-like proteins from different bacteria.**

Dra, *Deinococcus radiodurans* RecJ-like (PDB ID: 8IOO); Sa1, *Staphylococcus aureus* SA0013 NrnA (PDB ID: 8IU7); Sa2, *Staphylococcus aureus* SA0013 (GdpP) (PDB ID: 5XSI); Bsu, *Bacillus subtilis* YtqI (PDB ID: 5J21); Mtu, *Mycobacterium tuberculosis* Rv2837c (CnpB) (PDB ID: 5JJU); Tma, *Thermotoga maritima* TM1595 (PDB ID: 5O1U); Msm, *Mycolicibacterium smegmatis* MC2 155 NrnA (PDB ID: 4LS9); Sha, *Staphylococcus haemolyticus* JCSC1435 NrnA (PDB ID: 3DEV); Bfr, *Bacteroides fragilis* YCH46 (PDB ID: 3W5W). Secondary structural elements are depicted according to the structure of DrRecJ-like protein characterized in this study and displayed at the top of sequences.

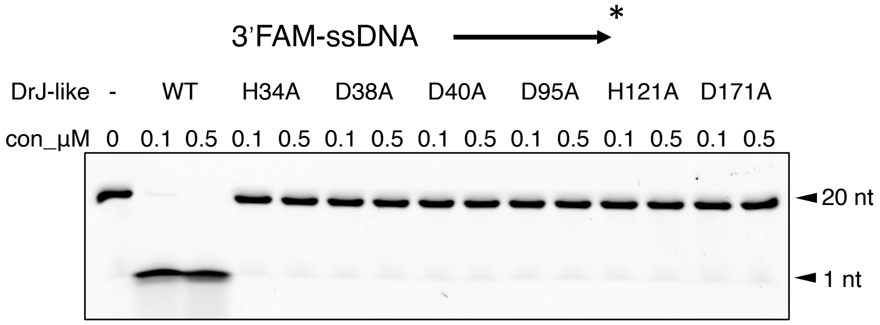


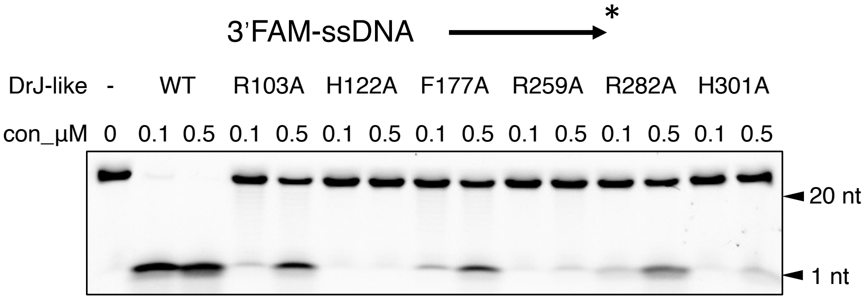


**Supplementary figure S6. Comparison of the digestion activities of different point mutants.**

Denaturing PAGE gel showing the reduced nuclease activity of point mutated drRecJ-like proteins (alanine substitutions of key residues involved in metal ion coordination or substrates binding). 3' FAM-labeled 20 nt ssDNA (200 nM) was incubated with different concentrations of DrRecJ-like proteins (0, 0.1 and 0.5 µM) in the presence of 1 mM $Mn^{2+}$ (see methods).
