# Supplementary material for: Structural and functional investigation of the DHH/DHHA1 family proteins in Deinococcus radiodurans
Source: Nucleic Acids Res. 2024 May 28;52(12):7142–57. doi: 10.1093/nar/gkae451 (PMC11229311; doi:10.1093/nar/gkae451)
Supplement: gkae451_Supplemental_Files [file gkae451_supplemental_files.zip › Table S3 legend.docx]

**Table S3 Differential gene expressions identified through transcript level analysis of RNA-seq data.**

The genes exhibiting obvious differences in transcript level between the *△J* and WT strains, the *△J-like* and WT strains, as well as the *△J* /*△J-like* and WT strains, were listed and described. Gene_id1, gene ID from RNA seq results; gene_id2, gene ID from KEGG annotation.
